# The architecture of resilience: a genome assembly of *Myrothamnus flabellifolia* sheds light on desiccation tolerance and sex determination

**DOI:** 10.1111/nph.70700

**Published:** 2025-11-02

**Authors:** Rose A. Marks, John T. Lovell, Sarah B. Carey, Llewelyn Van Der Pas, Nyaradzai M. Chimukuche, Tomáš Brůna, Christopher Plott, Jenell Webber, Anna Lipzen, Juying Yan, Diane Bauer, Joanne Bentley, Jayson Talag, Chloee M. McLaughlin, Kerrie Barry, Jane Grimwood, Jerry W. Jenkins, Jeremy Schmutz, Alex Harkess, Robert VanBuren, James Leebens‐Mack, Jill M. Farrant

**Affiliations:** ^1^ Department of Plant Biology University of Illinois Urbana IL 61801 USA; ^2^ Plant Resilience Institute Michigan State University East Lansing MI 48824 USA; ^3^ Department of Molecular and Cell Biology University of Cape Town Rondebosch 7700 South Africa; ^4^ HudsonAlpha Institute for Biotechnology Huntsville AL 35806 USA; ^5^ US Department of Energy Joint Genome Institute Lawrence Berkeley National Laboratory Berkeley CA 94720 USA; ^6^ African Climate and Development Initiative University of Cape Town Rondebosch 7700 South Africa; ^7^ Arizona Genomics Institute University of Arizona Tucson AZ 85721 USA; ^8^ Department of Plant Biology Michigan State University East Lansing MI 48824 USA; ^9^ Department of Plant, Soil, & Microbial Sciences Michigan State University East Lansing MI 48824 USA; ^10^ Department of Plant Biology and The Plant Center University of Georgia Athens GA 30602 USA

**Keywords:** chromosome structure, desiccation tolerance, drought, genome architecture, genomics, resurrection plants, sex determination, transcriptomics

## Abstract

- *Myrothamnus flabellifolia* is a dioecious resurrection plant endemic to southern Africa that has become an important model for understanding desiccation tolerance. Despite its ecological and medicinal significance, genomic and transcriptomic resources for the species are limited.
- We generated a chromosome‐level, haplotype‐resolved reference genome assembly and annotation for *M. flabellifolia* and conducted transcriptomic profiling across a natural dehydration–rehydration time course in the field. Genome architecture and sex determination were characterized, and co‐expression network and *cis*‐regulatory element (CRE) enrichment analyses were used to investigate dynamic responses to desiccation.
- The 1.28‐Gb genome exhibits unusually consistent chromatin architecture with unique chromosome organization across highly divergent haplotypes. We identified an XY sexual system with a small sex‐determining region on Chromosome 8. Transcriptomic responses varied with dehydration severity, pointing to early suppression of growth, progressive activation of protective mechanisms, and subsequent return to homeostasis upon rehydration. Late embryogenesis abundant and early light‐induced protein transcripts were dynamically regulated and showed enrichment of abscisic acid and stress‐responsive CREs pointing toward conserved responses.
- Together, this study provides foundational resources for understanding the genomic architecture and reproductive biology of *M. flabellifolia* and offers new insights into the mechanisms of desiccation tolerance.

## Introduction

Plants have evolved diverse and elegant adaptations to survive drought. Understanding these adaptations and the mechanisms underlying them is important for addressing the challenges associated with water scarcity. Among these, desiccation tolerance–the ability to dry to a quiescent state and resume normal cellular function when rehydrated–is one of the most remarkable (Marks *et al*., 2025). Desiccation tolerance has played an important role in the evolution of terrestrial plants, enabling the colonization of land (Oliver *et al*., 2000, 2005; Farrant & Moore, 2011; Zhang *et al*., 2020) and eventually the domestication of crops through seed storage. Unlocking the molecular, cellular, and physiological mechanisms of desiccation tolerance could facilitate transformative innovations in agriculture, medicine, and material sciences, such as advanced xeropreservation techniques that enhance survival in dry conditions (Marks *et al*., 2025). As global droughts intensify, understanding these mechanisms is critical for safeguarding agricultural production, biodiversity conservation, and the communities that rely on these resources.

Desiccation tolerance has evolved repeatedly and convergently across diverse plant lineages (Oliver *et al*., 2000; VanBuren *et al*., 2019; Alejo‐Jacuinde *et al*., 2020; Marks *et al*., 2024), but its mechanisms remain incompletely understood and overlap considerably with more generalized responses to water limitation in plants (Pardo *et al*., 2020). The so‐called resurrection plants exemplify vegetative desiccation tolerance (Gaff, 1971; Gaff & Hallam, 1974; Griffiths *et al*., 2014; Tebele *et al*., 2021; Marks, 2024) and can survive months to years in a dry state, often under intense solar radiation and high heat. Remarkably, they still resume full functionality within hours of rehydration (Oliver *et al*., 2020; Marks *et al*., 2021). This ability relies on complex molecular and physiological mechanisms that preserve cellular integrity during desiccation and facilitate recovery upon rehydration (Giarola *et al*., 2017; Oliver *et al*., 2020; Gechev *et al*., 2021). Key components of these survival mechanisms include the accumulation of osmoprotectants, such as nonreducing sugars and polyols that stabilize proteins and membranes in a dry state (Oliver *et al*., 2011; Holzinger & Karsten, 2013; Vieira *et al*., 2017); the production of late embryogenesis abundant (LEA) proteins (Hernández‐Sánchez *et al*., 2022) and early light‐induced proteins (ELIPs) (VanBuren *et al*., 2019) that mitigate photooxidative damage; and the activation of antioxidant pathways that scavenge reactive oxygen species (ROS) (Dinakar *et al*., 2012). Vegetative desiccation tolerance is also associated with the downregulation of photosynthesis and growth‐related pathways (Challabathula *et al*., 2018), the modification of cell walls to increase flexibility (Moore *et al*., 2006, 2023; Neeragunda Shivaraj *et al*., 2018; Chen *et al*., 2019; Plancot *et al*., 2019; Alejo‐Jacuinde *et al*., 2020), and the mobilization of proteostasis mechanisms, such as ubiquitination and autophagy (Zhu *et al*., 2015; Hibshman *et al*., 2023; VanBuren *et al*., 2023) that help to repair cellular components upon rehydration (Oliver *et al*., 2020). Taken together, dynamic changes in gene expression drive metabolic reprogramming, cellular protection, and repair processes that enhance survival during desiccation (Alejo‐Jacuinde *et al*., 2020; Pardo *et al*., 2020; VanBuren *et al*., 2023; Marks *et al*., 2024; Zhang *et al*., 2024). Upon rehydration, these mechanisms are reversed or modified to support recovery and resume normal physiological functions (Oliver *et al*., 2020).

*Myrothamnus flabellifolia Welw* is one of the most iconic resurrection plants, having captured the attention of local communities and scientists for decades (Moore *et al*., 2007; Marks *et al*., 2022). It is locally abundant in some of the harshest environments in southern Africa, occupying a unique niche on rocky outcrops and cliffs. The species' striking resilience is associated with the production of a suite of specialized compounds, including antioxidants, phenolics, and other diverse secondary metabolites, many of which have traditional and emerging medicinal significance (Bentley *et al*., 2019). Additionally, *M. flabellifolia* is dioecious, which provides a rare opportunity to study the evolution of sex determination and sexual dimorphisms in the context of extreme stress tolerance (Marks *et al*., 2022). Lastly, *Myrothamnus* (Myrothamnaceae) and *Gunnera* (Gunneraceae) are the only two extant genera within the order Gunnerales, which is the sister lineage to the hyperdiverse Pentapetalae clade including all other core eudicots (Gunneridae) (Wanntorp *et al*., 2001; Cantino *et al*., 2007). These features position *M. flabellifolia* as a key system for exploring desiccation tolerance, evolutionary biology, and medicinal applications.

Here, we present a chromosome‐level, haplotype‐resolved reference genome assembly and annotation for *M. flabellifolia*, coupled with a high‐resolution transcriptomic characterization of a natural dehydration and rehydration event in the field. By leveraging both genomic and transcriptomic resources, we explore the mechanisms underlying dioecy and sex determination, vegetative desiccation tolerance, and comment on the medicinal implications of the specialized metabolism of *M. flabellifolia*.

## Materials and Methods

### Study organism

**Fig. 1:**
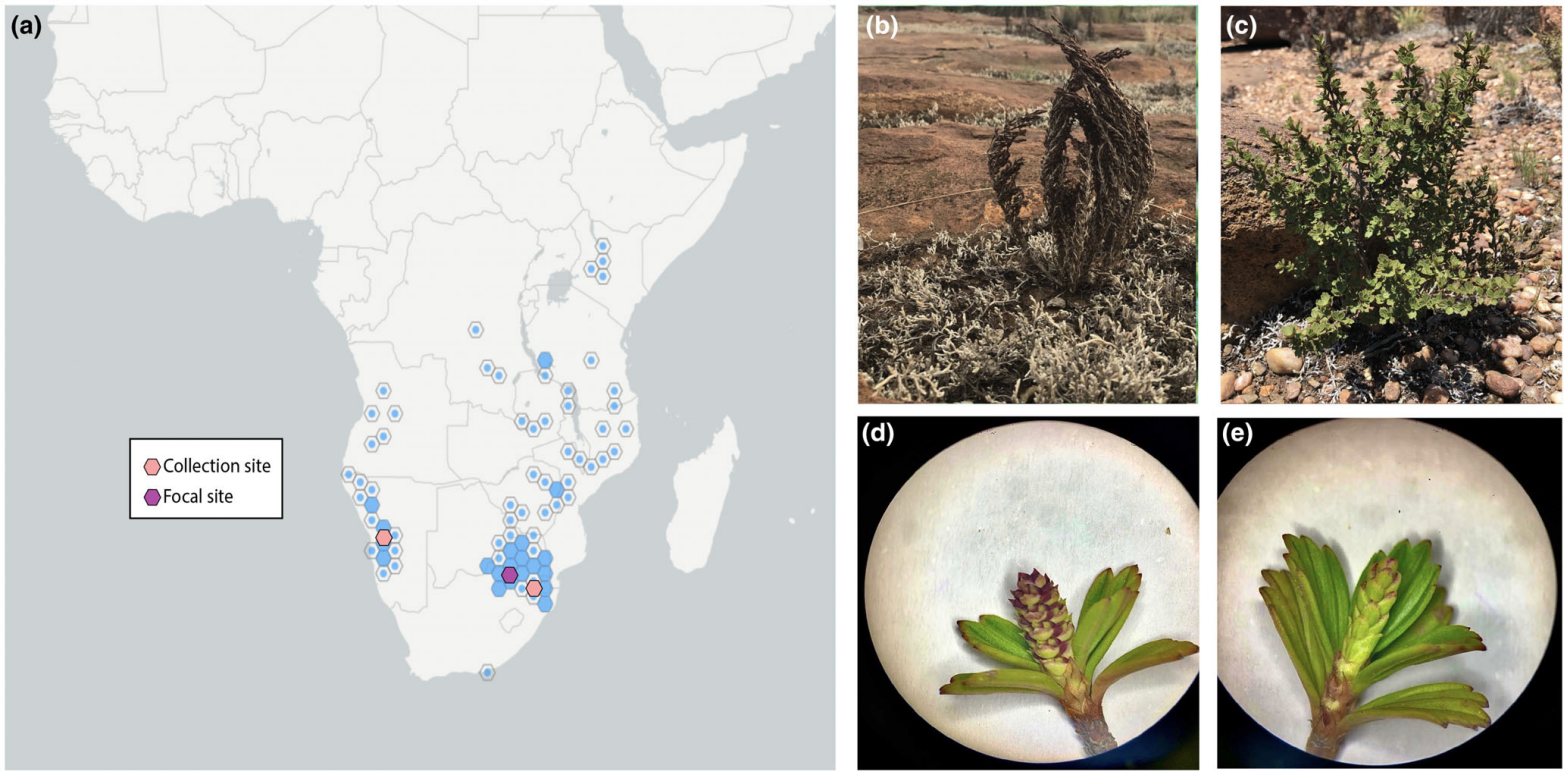
Distribution and anatomy of *Myrothamnus flabellifolia*. (a) Geographic distribution of *M. flabellifolia* across southern Africa. Blue hexagons indicate the overall distribution of *M. flabellifolia* at a coarse spatial resolution. Study sites for the current work are highlighted, with the focal site in Limpopo, South Africa, shown in darker purple and additional sites in Namibia and Mpumalanga, South Africa, shown in light pink. Representative plants in their native habitat are shown in both (b) desiccated and (c) hydrated conditions. Floral morphology of (d) male and (e) female plants.

*Myrothamnus flabellifolia Welw* is a dioecious resurrection plant in the eudicot lineage, Gunnerales. Myrothamnaceae contains only one genus with just two species in it: *M. flabellifolia* and *M. moschatus*. *Myrothamnus moschatus* is endemic to Madagascar and *M. flabellifolia* is distributed throughout southern Africa in disjunct populations (Fig. 1). Both species occupy a narrow ecological niche, restricted to rocky sites, with minimal soil, intense abiotic stresses (e.g. aridity, heat, and irradiation), and low competition. *Myrothamnus* is unique among resurrection plants as it is the only dioecious angiosperm resurrection plant, is large and woody growing up to *c*. 1.5 m tall, and produces a robust profile of secondary compounds with important cultural history and medicinal applications.

### Field sites and sample collections

We collected *M. flabellifolia* plants for this study from three distinct sites in southern Africa. These sites span substantial geographic distance and environmental variation, from extremely arid sites in Erongo, Namibia (−22.00922 S, 15.92595 E), to intermediate sites in Limpopo, South Africa (−23.7949, 28.0705), to mesic sites in Mpumalanga, South Africa (−25.30229 S, 30.50631 E). We marked the locations of study sites with a Garmin 64csx GPS, tagged target plants, and collected leaf tissue for downstream analyses. We selected a single focal site in Limpopo, South Africa, for high‐resolution transcriptomic profiling across a dehydration time course.

Initially, we sampled 10 plants across the three study sites. We collected leaf tissues from two male and two female plants each in Mpumalanga and Limpopo, South Africa. We collected leaf tissue from one male and one female from the site in Namibia. Dry tissue from each plant was shipped to the United States under permit no. P37‐19‐01892 and according to the material transfer agreement (MTA) established between Drs Farrant, VanBuren, and Marks. Leaf tissues were rehydrated, harvested, and flash‐frozen in liquid nitrogen. Genomic DNA was extracted and processed for Illumina DNAseq as described in detail later. The resulting sequence data were analyzed to estimate diversity across populations, heterozygosity, and to identify the sex determination region of *M. flabellifolia* (described in detail later).

Based on these analyses, we targeted a single male plant from Limpopo South Africa for reference genome sequencing and assembly. We selected a male plant because our analysis indicated that *M. flabellifolia* has an XY sex determination system and sequencing the heterogametic sex would thus provide a more comprehensive assessment of the genome sequence. For the reference genome assembly, we collected additional healthy green tissue from the reference accession (var. SSDT_37) in the field and immediately flash‐frozen it in liquid nitrogen. We shipped the frozen leaf tissue to the United States under United States Department of Agriculture permit no. P37‐19‐01892 and according to the MTA established between Drs Farrant, VanBurnen, and Marks.

### DNA extraction and library preparation

We extracted high‐molecular‐weight (HMW) DNA from the 10 accessions and the reference genotype (var. SSDT_37) following standard protocols. In short, we used the protocol of Doyle & Doyle (1987) with minor modifications. Flash‐frozen biomass was ground to a fine powder in a frozen mortar with liquid nitrogen followed by very gentle extraction in 2% cetyltrimethylammonium bromide (CTAB) buffer (that included proteinase K, PVP‐40, and beta‐mercaptoethanol) for 30 min to 1 h at 50°C. After centrifugation, the supernatant was transferred to a new tube, treated with 200 μl 50 mM phenylmethylsulfonyl fluoride (PSMF) for 10 min at room temperature then gently extracted twice with 24 : 1 chloroform : isoamyl alcohol. The upper phase was transferred to a new tube and one‐tenth volume of 3 M sodium acetate was added, gently mixed, and DNA precipitated with iso‐propanol. The DNA precipitate was collected by centrifugation, washed with 70% ethanol, air‐dried for 5–10 min, and dissolved thoroughly in an elution buffer at room temperature followed by RNAse treatment. DNA purity was measured with Nanodrop, DNA concentration measured with Qubit HS kit (Invitrogen), and DNA size was validated by Femto Pulse System (Agilent).

Illumina sequencing libraries were prepared by shearing 500 ng to 1.5 μg HMW DNA on a Covaris instrument to 350 bp. DNA fragments were bead‐cleaned, end‐repaired, and size selected to remove large and small fragments. After adenylation, adaptors were ligated according to the Illumina TruSeq PCR‐Free DNA Library Prep Kit. Libraries were sequenced on an Illumina 6000 Instrument PE150 (Table S1). PacBio DNA sequencing libraries were prepared by shearing HMW DNA using a megaruptor shearing device (Diagenode, Seraing, Belgium), ligating SMRTbell adaptors (SMRTbell Prep Kit v.3.0), and sizing the final library using a BluePippin Instrument (SAGE Science, Beverly, MA, USA) at 10–50 kb. Omni‐C libraries were prepared by grinding frozen leaf tissue using a freezer mill followed by library construction using a Dovetail‐C kit (Cantata Bio, Scotte Valley, CA, USA) (Table S2).

### Illumina and PacBio genome sequencing

We sequenced the genome of *M. flabellifolia* using a whole‐genome shotgun sequencing strategy according to standard sequencing protocols. Sequencing reads were generated using Illumina and PACBIO platforms. Illumina and PACBIO reads were sequenced at the HudsonAlpha Genome Sequencing Center in Huntsville, AL. Illumina reads were sequenced using the Illumina NovoSeq6000 platform. PacBio Sequencing primer was annealed to the SMRTbell template library, and sequencing polymerase was bound to them using Sequel II Binding kit 2.0. The prepared SMRTbell template library was then sequenced on a Pacific Biosciences Sequel IIe sequencer using 8 M v.1 SMRT cells and v.2.0 sequencing chemistry with 1×1800 sequencing movie run times. Two 400‐bp insert 2×150 Illumina fragment libraries were sequenced with a coverage of 89.28× along with one 2×150 Hi‐C library with a coverage of 56.69× (Table S1) sequenced on a NovaSeq 6000 Instrument to generate 150‐bp paired‐end reads. Before assembly, Illumina fragment reads were screened for phix contamination. Reads composed of > 95% simple sequence were removed. Illumina reads < 50 bp after trimming for adapter and quality (*q* < 20) were removed. The final read set consisted of 1 592 906 904 reads for a total of 89.28× coverage of high‐quality Illumina bases. For the PACBIO sequencing, a total raw sequence yield of 110.49 Gb, with a total coverage of 42.99× per haplotype, was generated (Table S2).

### Genome assembly and construction of pseudomolecule chromosomes

Haplotype 1 (HAP1) and HAP2 v.1.0 assemblies were generated by assembling the 6672 299 PACBIO CCS reads (42.99× per haplotype) using the HiFiAsm+HIC assembler (Cheng *et al*., 2021) and subsequently polished using RACON (Vaser *et al*., 2017). This produced initial assemblies of both haplotypes. The HAP1 assembly consisted of 370 scaffolds (370 contigs), with a contig N50 of 13.6 Mb, and a total genome size of 1290.4 Mb (Table S3). The HAP2 assembly consisted of 333 scaffolds (333 contigs), with a contig N50 of 10.9 Mb, and a total genome size of 1295.2 Mb (Table S4).

To improve these initial assemblies, Hi‐C Illumina reads from *M. flabellifolia* reference accession (var. SSDT_37), were separately aligned to the HAP1 and HAP2 contig sets with Juicer (Durand *et al*., 2016), and chromosome‐scale scaffolding was performed with 3D‐DNA (Dudchenko *et al*., 2017). No misjoins were identified in either the HAP1 or HAP2 assemblies. The contigs were then oriented, ordered, and joined together into 10 chromosomes per haplotype using a combination of the Hi‐C read alignments and the JuiceBox contact map viewer. Contigs containing significant telomeric sequences were properly oriented in the assembly. In‐house tools were used to identify redundant contigs. A total of 154 joins were applied to the HAP1 assembly, and 179 joins for the HAP2 assembly. Each chromosome join was padded with 10 000 Ns. Contigs terminating in significant telomeric sequences were identified using the (TTTAGGG)_*n*_ repeat, and care was taken to make sure that they were properly oriented in the production assembly. The remaining scaffolds were screened against bacterial proteins, organelle sequences, GenBank nr, and removed if found to be contaminants. After forming the chromosomes, it was observed that some small (< 20 kb) redundant sequences were present on adjacent contig ends within chromosomes. To resolve this issue, adjacent contig ends were aligned to one another using BLAT (Kent, 2002), and duplicate sequences were collapsed to close the gap between them. A total of nine adjacent contig pairs were collapsed in the HAP1 assembly and three in the HAP2 assembly. The *M. flabellifolia* chloroplast and mitochondrial genomes were assembled with the OatK pipeline (https://github.com/c‐zhou/oatk) utilizing the angiosperm database. The chloroplast and mitochondrial genomes were then polished using RACON (Vaser *et al*., 2017) and included as part of the release.

Finally, homozygous single nucleotide polymorphisms (SNPs) and insertions / deletions (INDELs) were corrected in the HAP1 and HAP2 releases using *c*. 54× of Illumina reads (2×150, 400 bp insert) by aligning the reads using Burrows‐Wheeler Aligner ‐ Maximum Exact Matches (BWA‐MEM) (Li, 2013) and identifying homozygous SNPs and INDELs with the Genome Analysis Toolkit (GATK's) UnifiedGenotyper tool (McKenna *et al*., 2010). A total of 920 homozygous SNPs and 9867 homozygous INDELs were corrected in the HAP1 release, while a total of 862 homozygous SNPs and 10 258 homozygous INDELs were corrected in the HAP2 release. The final v.1.0 HAP1 release contained 1285.3 Mb of sequence, consisting of 195 contigs with a contig N50 of 11.4 Mb and a total of 99.99% of assembled bases in chromosomes (Table S5). The final v.1.0 HAP2 release contains 1274.7 Mb of sequence, consisting of 169 contigs with a contig N50 of 13.7 Mb and a total of 100% of assembled bases in chromosomes (Table S6).

Completeness of the euchromatic portion of the v.1.0 assemblies was assessed using an rnaSEQ library (library ID: HOSHY). The aim of this analysis is to obtain a measure of completeness of the assembly, rather than a comprehensive examination of gene space. The transcripts were aligned to the assembly using BWA‐MEM (Li, 2013). The screened alignments indicate that 98.89% of the rnaSEQ reads aligned to the HAP1 v.1.0 release, and 98.89% aligned to the HAP2 v.1.0 release.

SyRI (Goel *et al*., 2019) was used for pairwise whole‐genome alignment between the two Myrothamnus haplotypes (HAP1 and HAP2) with minimap2 (Li, 2018) variant detection. Pairwise alignment files were generated in minimap2 using default parameters and a divergence parameter of asm5, which is appropriate for within‐species comparisons. Differences in the pairwise alignment file between the HAP1 and HAP2 genomes were annotated with SyRI.

### Genome annotation

Transcript assemblies were made from *c*. 455 M pairs of 2×150 stranded paired‐end Illumina RNA‐seq reads using PERTRAN, which conducts genome‐guided transcriptome short‐read assembly via GSNAP v.2019‐09‐12 (Wu & Nacu, 2010) and builds splice alignment graphs after alignment validation, realignment, and correction. To obtain *c*. 663 000 and *c*. 664 000 (for HAP1 and HAP2, respectively) putative full‐length transcripts, *c*. 15 M PacBio Iso‐Seq circular consensus sequencing (CCSs) reads were corrected and collapsed by a genome‐guided correction pipeline. The pipeline aligns CCS reads to the genome with GMAP v.2019‐09‐12 (Wu & Nacu, 2010), corrects small indels in splice junctions, and clusters alignments when all introns are the same or ≥ 95% overlap for single‐exon alignments. Subsequently, PASA v.2.0.2 (Haas *et al*., 2003) was used to construct 519 584 and 517 780 (for HAP1 and HAP2, respectively) transcript assemblies by combining the transcript assembly sets described previously.

A repeat library was created from *de novo* repeats predicted by repeatmodeler2 v.2.0.4 (Flynn *et al*., 2020) on the *M. flabellifolia* var. *SSDT_37* HAP1 v.1.0 genome. The predicted repeats underwent functional analysis through interproscan v.5.26‐65.0 (Jones *et al*., 2014), incorporating the Pfam (Mistry *et al*., 2021) and PANTHER (Mi *et al*., 2019) databases. Any repeats that displayed significant hits to protein‐coding domains were subsequently excluded from the repeat set. Finally, the constructed species‐specific repeat library was used to soft‐mask both haplotypes with repeatmasker v.4.1.2 (Flynn *et al*., 2020). We additionally annotated repeats for both HAP1 and HAP2 genome assemblies using panEDTA in EDTA2 v.2.2.1 (Ou *et al*., 2019, 2024) with default parameters.

Putative gene loci were determined by transcript assembly alignments and/or EXONERATE v.2.4.0 (Slater & Birney, 2005) alignments of proteins from *Mimulus guttatus*, *Lactuca sativa*, *Hydrangea quercifolia*, *Kalanchoe fedtschenkoi*, *Arabidopsis thaliana*, *Vitis vinifera*, *Gossypium hirsutum*, *Populus trichocarpa*, *Glycine max*, *Prunus persica*, *Liriodendron tulipifera*, *Eschscholzia californica*, *Solanum lycopersicum*, *Beta vulgaris*, *Brassica rapa*, *Citrus sinensis*, *Medicago truncatula*, *Sorghum bicolor*, *Oryza sativa*, *Osyris compressa*, *Macadamia integrifolia*, *Telopea speciosissima*, *Nelumbo nucifera*, and Swiss‐Prot release 2022_04 of eukaryotic proteomes to repeat‐soft‐masked *M. flabellifolia* var. *SSDT_37* v.1.0 HAP1 and HAP2 genomes with up to 2000‐bp extension on both ends unless extending into another locus on the same strand. Gene models in each locus were predicted by homology‐based predictors, fgenesh+ v.3.1.1 (Salamov & Solovyev, 2000), FGENESH_EST (similar to FGENESH+, but using EST to compute splice site and intron input instead of protein/translated ORF), EXONERATE, PASA assembly ORFs (in‐house homology constrained open reading frame (ORF) finder), and augustus v.3.3.3 (Stanke *et al*., 2006) trained on the high confidence PASA assembly ORFs and with intron hints from short‐read alignments. The best‐scored predictions for each locus were selected using multiple positive factors including expressed sequence tags (ESTs) and protein support, and one negative factor: overlap with repeats. The selected gene predictions were improved by PASA. The improvement included adding untranslated regions (UTRs), splicing correction, and adding alternative transcripts.

PASA‐improved gene model proteins were subjected to protein homology analysis to the previously mentioned proteomes to obtain a Cscore and protein coverage. Cscore is a protein blastp score ratio to the mutual best hit blastp score, and protein coverage is the percentage of protein aligned to the best of homologs. PASA‐improved transcripts were selected based on Cscore, protein coverage, EST coverage, and their coding sequences (CDS) overlap with repeats. The transcripts were selected if their Cscore and protein coverage were ≥ 0.5 or if covered by ESTs. For gene models whose CDS overlapped repeats by > 20%, their Cscore had to be at least 0.9 and homology coverage at least 70% to be selected. The selected gene models were subject to Pfam analysis and gene models without strong transcriptome and homology support whose proteins were > 30% overlapped by Pfam transposable element (TE) domains were removed. Incomplete gene models, low homology supported without fully transcriptome‐supported gene models, short single exon (< 300 bp CDS) without protein domains nor good expression, and repetitive gene models without strong homology support were manually filtered out.

TRASH (Wlodzimierz *et al*., 2023) was used to annotate repetitive sequences for the identification of putative centromeric regions in *M. flabellifolia*. Ultimately, we did not observe distinct clustering of satellite sequences and instead found satellite repeats to be distributed across the genome assemblies for both HAP1 and HAP2. The most common repeat annotated by TRASH was a 7‐bp satellite that occurs regularly and is widespread across the genome. Given that this 7‐bp satellite is so widespread, we additionally checked for any structure of the next most frequent satellites identified by TRASH. For the next eight most frequent satellites identified by TRASH (26 bp, 34 bp, 35 bp 49 bp, 63 bp, 64 bp, 65 bp, and 67 bp), we did not observe any obvious signals of clustering and the distribution of satellite sequences was spread around the genome.

### Sex determination region

We used Illumina whole‐genome resequence data from 10 *M. flabellioflia* accessions (five females, five males) to identify k‐mers unique to males (Y‐mers; Carey *et al*., 2024a,b). We filtered the sequence data using trimmomatic v.0.39 with leading and trailing values of three, a sliding window of 30, a jump of 10, and a minimum remaining read length of 40 (Bolger *et al*., 2014) and identified k‐mers using meryl v.1.3 *count* (Rhie *et al*., 2020). We used meryl's *intersect* function to identify all 21‐mers shared in females, followed by *difference* to identify the 21‐mers only found in males. Y‐mers were mapped to the assemblies in order to identify putatively Y‐associated contigs using bwa‐mem v.0.7.17 (Li & Durbin, 2009; Li, 2013) with flags ‘‐k 21’, ‘‐T 21’, ‘‐a’, and ‘‐c 10’. To determine which genes were X‐ or Y‐specific, we used orthofinder v.2.5.2 (Emms & Kelly, 2015, 2019), to identify homologs.

### Field sampling and physiological measurements across desiccation time course

We tracked 12 plants from the focal population in Limpopo South Africa across a natural dehydration–rehydration event in the field. All plants were located within a single *c*. 300 × 100 m patch at Swebe Swebe and therefore experienced the same rainfall events, although differences in topography, soil depth, and canopy cover likely influenced the rate of water loss in each individual. Study plants were tagged and phenotyped to quantify height (*c*. 60–100 cm to ensure comparable maturity and hydraulic dynamics) and sex (equal numbers of males and females) (Marks *et al*., 2022). Plants were spaced at least 3 m apart to ensure that each was a distinct individual. These plants were then monitored throughout a natural dehydration and rehydration event spanning 6 d in the field. Each plant was sampled at baseline hydrated conditions (14:00 h on 14 January 2020) and at six progressive dehydration time points. Dehydration sampling was made at 44 h (10:00 h, 16 January 2020), 48 h (14:00 h, 16 January 2020), 52 h (18:00 h, 16 January 2020), 68 h (10:00 h, 17 January 2020), 72 h (14:00 h, 17 January 2020), and 76 h (18:00 h, 17 January 2020) after sampling began. We then waited for a natural rainfall event to occur and sampled rehydration time points at 2 h (09:00 h, 18 January 2020), 4 h (11:00 h, 18 January 2020), 8 h (15:00 h, 18 January 2020), 12 h (19:00 h, 18 January 2020), 24 h (09:00 h, 19 January 2020), and 48 h (09:00 h, 20 January, 2020) after rainfall. Environmental data on temperature and relative humidity were tracked with a Hobo weather station and U23 pro V2 dataloggers. At each sampling time point, we quantified the relative water content (RWC) of the sample and flash‐frozen leaf tissue in liquid nitrogen for downstream RNA extractions. RWC was quantified by measuring the mass of 10–15 leaves from each plant. Leaf mass was weighed immediately after collection (fresh weight), again after 48 h submerged in *d*H_2_0 in darkness at 4°C (turgid weight), and finally after 48 h in a 70°C drying oven (dry weight). RWC was calculated as (fresh weight–dry weight)/(turgid weight–dry weight). Over the course of the experiment, individual plants dried at different rates, and we assigned them to groups based on how much they dried during the time course. Plants were assigned to either ‘mild dehydration’, ‘severe dehydration’, or ‘desiccation’ groups according to their minimum RWC during the time course.

### RNA extraction and sequencing

RNA was extracted from a total of 149 samples from 12 individuals across 13 time points. Three additional samples from flower tissue were included to aid in genome annotation. RNA was extracted using the Spectrum total plant RNA kit according to the manufacturer's instructions, with on‐column DNAse treatment included in the pipeline. RNA samples were further processed to remove impurities and contaminants using the Zymo RNA clean and concentrate kit according to the manufacturer's instructions.

Plate‐based RNA library prep was performed on the PerkinElmer Sciclone NGS robotic liquid handling system using Illumina's TruSeq Stranded mRNA HT sample prep kit utilizing poly‐A selection of mRNA following the protocol outlined by Illumina in their user guide (https://support.illumina.com/sequencing/sequencing_kits/truseq‐stranded‐mrna.html). Briefly, total RNA starting material was 1000 ng per sample and eight cycles of PCR were used for library amplification. The prepared libraries were quantified using KAPA Biosystems' next‐generation sequencing library quantitative polymerase chain reaction kit and run on a Roche LightCycler 480 real‐time PCR instrument. Sequencing of the flowcell was performed on the Illumina NovaSeq sequencer using NovaSeq XP V1.5 reagent kits, S4 flowcell, following a 2×151 indexed run recipe. RNA libraries were sequenced on an Illumina NovaSeq S4 for 150 bp PE reads.

Long‐read RNA‐seq (isoSeq) data were generated for 12 leaf and flower samples from the reference accession (var. SSDT_37) to aid in genome annotation. Full‐length cDNA was synthesized using template switching technology with the NEBNext Single Cell/Low Input cDNA Synthesis & Amplification Module kit. The first‐strand cDNA was amplified and multiplexed with NEBNext High‐Fidelity 2× PCR Master Mix using barcoded cDNA PCR primers. The amplified cDNA was purified using 1.3× ProNex beads for nonsize selection or 0.89× ProNex beads for above 2‐kb size selection and like sizes were pooled at equimolar ratios in a designated degree‐of‐pool in the worksheet using the PacBio Multiplexing Calculator. The pooled samples were end‐repaired, A‐tailed, and ligated with overhang nonbarcoded adaptors using the SMRTbell Express 2.0 kit. IsoSeq libraries were sequenced on a PacBioSequel II.

### Transcriptomic analyses

The resulting RNA‐seq reads were processed to quantify transcript abundance following a pipeline developed by the VanBuren Lab (https://github.com/pardojer23/RNAseqV2). Briefly, read quality was assessed with fastqc (v.0.23), and reads were trimmed with trimmomatic (v.0.38) (Bolger *et al*., 2014) to remove adapters and low‐quality bases. Trimmed reads were sudoaligned to the reference genome using salmon (v.1.9.0) (Patro *et al*., 2017), and the resulting quantification files were processed with tximport (v.3.18) (Soneson *et al*., 2015) to generate raw count and transcripts per million (TPM) expression matrices. Hierarchical clustering was conducted for basic quality control and visualization of sample relationships across experimental time points and biological replicates. A principal component analysis (PCA) of TPM values was used to further visualize replicate and sample relationships.

While PCA provided some level of dimensionality reduction, residual heterogeneity, experimental differences, noise, or genotype‐level differences in the dataset might have obscured underlying biology. To address this, we applied topological data analysis (TDA) using the Mapper algorithm (van Veen *et al*., 2019; Palande *et al*., 2023; Marks *et al*., 2024), which provides a flexible and scalable approach for exploring high‐dimensional, sparse datasets. Mapper requires a lens function, a user‐defined feature that shapes how the data are clustered and connected. We used RWC as the lens, anchoring it to the fully hydrated condition, to reflect physiological water status across samples. This allowed us to explore expression changes along a continuum of dehydration and recovery. For our mapper graph, we specified 110 intervals with a 90% overlap. The topology was plotted to visualize relationships across samples.

Differentially abundant transcripts (DATs) were identified with the deseq2 v.1.42.0 (Love *et al*., 2014) defined as Log_2_FC > |2| and Bonferroni adjusted *P*‐values < 0.05. Initially, we tested multiple models for identifying DATs in DEseq2, including a model that identified DATs by pairwise comparisons of each time point against hydrated samples and a model that used the continuous variable of RWC as a covariate. DATs identified by pairwise comparisons were summarized into a nonredundant list of up‐ and downregulated genes during dehydration and rehydration. To select the best‐performing model, we quantified similarities and differences in the number and identity of DATs defined by each model using Venn diagrams. There was a high degree of overlap in DATs identified by both models. Ultimately, we selected the model based on pairwise comparisons for downstream analyses because it provided differentiation between the effects of time and drying rate, whereas the model based on RWC did not account for the effect of time.

We identified DATs separately for plants in each of the three drying groups (fast, intermediate, and slow) and compared the overlap in DATs between groups using Venn diagrams. We then investigated the functional significance of the shared and unique DATs using Gene Ontology (GO) enrichment analysis (Bonferroni adjusted *P*‐values < 0.05) withTopGO (Rahnenfuhrer, 2019). We further visualized changes in DATs across the time course with Alluvial diagrams generated with the alluvial R package v.0.1‐2 (Edwards & Bojanowski, 2016).

While DAT analyses can be informative for identifying and describing overarching patterns and large shifts in transcript abundance, more nuanced patterns of gene expression and regulation can be obscured in classical differential analysis. To gain insight into these aspects, we generated co‐expression networks using the weighted gene co‐expression network analysis (WGCNA) R package v.1.7 (Langfelder & Horvath, 2008). Co‐expression modules were identified using the complete transcript abundance matrix of all genes in all samples. To construct the co‐expression network, we first determined a soft thresholding power for the dataset. The thresholding power was chosen to satisfy WGCNA's assumption that a weighted co‐expression network is scale‐free. An adjacency matrix, representing the strength of connections between genes in the network, was constructed using the selected soft thresholding power. This matrix was then converted to a topological overlap matrix (TOM), and hierarchal clustering was used on the TOM to group genes into modules based on similar expression patterns. We plotted the expression profiles of each module for each of the drying groups separately. This allowed us to visualize differences between the drying groups for each gene expression module. We then investigated the functional significance of modules using GO enrichment analysis, implemented with the R package topgo (Rahnenfuhrer, 2019) as described previously.

Both LEA and ELIP gene families have important roles in desiccation tolerance (VanBuren *et al*., 2019; Hernández‐Sánchez *et al*., 2022), and we conducted targeted analyses to quantify the expression dynamics of these gene families. LEA proteins were identified by the genome annotation pipeline described previously, and ELIPs were identified via a blast search against the Arabidopsis ELIP1 gene. We plotted the normalized expression of each LEA and ELIP paralog in each sample to visualize expression patterns across the time course in heatmaps. The predicted peptide sequences of each LEA and ELIP transcript were analyzed on expasy (Gasteiger *et al*., 2003) to retrieve characterization information and on pondr (Xue *et al*., 2010) for disorder prediction. clustalomega (Sievers *et al*., 2011) was used to generate multiple sequence alignments of sequences and to retrieve percent identity per protein group using default settings. We calculated pairwise correlations between peptide features of different LEAs and ELIPs using a Pearson's *r* correlation matrix implemented in pandas.

To investigate the *cis*‐regulatory landscape of LEA gene expression during dehydration in *M. flabellifolia*, we first extracted the 1‐kb upstream promoter regions of all LEA genes identified in co‐expression modules. Promoter coordinates were determined using the annotated GFF3 file for the *M. flabellifolia* (var. SSDT_37) genome, and strand orientation was taken into account to extract the appropriate upstream regions using custom awk commands. Promoter sequences were retrieved from the reference genome using bedtools v.2.30.0 (Quinlan & Hall, 2010). *Cis*‐regulatory elements (CREs) within these promoter regions were then identified using the PLACE v.30.0 database (Higo *et al*., 1999). Each CRE's presence or absence was recorded as a binary matrix, in which rows represented LEA genes and columns represented individual CREs. LEA genes were grouped based on their co‐expression module assignment: Turquoise, magenta, and cyan modules were categorized as upregulated during dehydration, while black, blue, and pink modules were categorized as downregulated.

To determine whether specific CREs were enriched in the promoters of up‐ or downregulated LEA genes, we performed logistic regression analysis using R. Each CRE was tested individually for its association with gene expression group (upregulated vs downregulated) using a binomial generalized linear model. The dependent variable was module group (downregulated vs upregulated), and the independent variable was CRE presence vs absence. Odds ratios and *P*‐values were extracted for each CRE. Odds ratios > 1 indicate enrichment in upregulated genes, while odds ratios less than 1 indicate enrichment in downregulated genes. CREs with *P*‐values < 0.05 were considered statistically significant. We used this information to identify regulatory motifs that may contribute to LEA transcript abundance patterns in response to dehydration.

## Results

### Study organism and accessions

*Myrothamnus flabellifolia* is an iconic resurrection plant in the eudicot lineage Gunnerales. Myrothamnaceae contains only one genus with just two species in it: *M. flabellifolia* and *M. moschatus* (Moore *et al*., 2007). *Myrothamnus moschatus* is endemic to Madagascar while *M. flabellifolia* is distributed throughout continental southern Africa in disjunct populations (Fig. 1a). Both species occupy a narrow ecological niche, restricted to rocky sites, with minimal soil, and intense abiotic stresses (Marks *et al*., 2022; Wan *et al*., 2024). In addition to being desiccation‐tolerant, *M. flabellifolia* is dioecious with separate male and female individuals (Fig. 1b–e). *M. flabellifolia* also produces a robust profile of secondary compounds with important traditional and emerging medicinal applications (Bentley *et al*., 2019). For this study, we collected *M. flabellifolia* plants from three populations across the species range (Fig. 1a) and generated a genome assembly for a single heterogametic (male) genotype from the selected focal site, Swebe Swebe Nature Reserve in Limpopo, South Africa. We also generated transcript abundance profiles for 12 genotypes during a natural dehydration–rehydration event (see the ‘Desiccation tolerance’ section) at Swebe Swebe Nature Reserve.

### The unusual genome structure of *M. flabellifolia*

We generated a chromosome‐level, haplotype‐resolved genome assembly for *M. flabellifolia* using deep PacBio HiFi sequencing technology (69.05× per haplotype; mean read length = 15 920 bp). The final genome assembly, which was scaffolded with 61.5× Hi‐C and polished with 54× Illumina reads, spans *c*. 1.28 Gb per haplotype, with HAP1 built from 195 contigs (*N*_50_ = 11.4 Mb) and HAP2 from 169 contigs (*N*_50_ = 13.7 Mb; Table 1). We annotated the assemblies with gene models supported by evidence derived from 12 RNA‐seq libraries (> 909 M total reads) constructed using RNAs extracted from tissues collected across five conditions and 12 Iso‐seq libraries constructed from pooled RNAs (> 15 M reads). The resulting annotation included 22 809/22 922 (HAP1/HAP2) protein‐coding gene models both with nearly perfect Eukaryote and ≥ 99% Embryophyta BUSCO scores.

**Table 1:** Genome assembly and gene annotation statistics for *Myrothamnus flabellifolia*.

	Haplotype 1	Haplotype 2
Chromosome sequence	1283.3 Mb	1273.1 Mb
Contig total	195	169
Scaffold *N*_50_	134.5 Mb	133.8 Mb
Contig *N*_50_	11.4 Mb	13.7 Mb
No. of genes/transcripts	22 809/47 534	22 922/47 563
Annotation BUSCO	99.0/98.7%	99.1/98.5
Annotation PSAURON	93.5	93.5

Statistics are reported by haplotype, but phasing was performed individually for each chromosome. *Chromosome sequence* indicates the total length of the assembled haploid genome. *Contig* and *Scaffold N*_50_ are measures of assembly completeness and refer to the length at which 50% of the assembly is contained in scaffolds or contigs of equal or greater size. *Annotation BUSCO* reflects the percentage of Benchmarking Universal Single‐Copy Orthologs from the embryophyta_odb10/eudicots_odb10 dataset that were captured, a measure of annotation completeness. *Annotation PSAURON* reports the proportion of predicted protein‐coding genes identified as high confidence by PSAURON, a computational tool that uses machine learning to assign likelihoods to protein annotations.

Comparisons of homologous chromosome assemblies between the two haplotypes revealed a high level of nucleotide diversity with an average of 47.38 SNPs per 10 kb. We also observed 459 (50.8 Mb total) large insertions, deletions, or inversions > 10 kb. Of particular note was a large inversion on Chr01 spanning 18.4 Mb (11.3% of the chromosome). Commensurate with this molecular variation, we observed significant gene content variation between the haplotypes – of the 22 902 total gene families, 2605 (11.3%) and 1254 (5.5%) exhibit presence–absence or copy number variation, respectively.

**Fig. 2:**
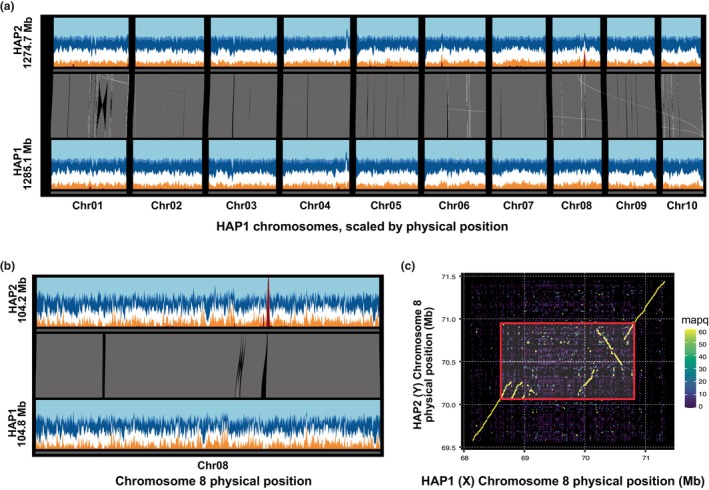
Unusual genome structure of *Myrothamnus flabellifolia*. (a) Genome‐wide synteny (gray links) shows that one large (Chr01) and several small inversions exist between the two haplotypes (HAP1 and HAP2) of the reference genome, but otherwise the vast majority of the sequences are syntenic and readily alignable. In contrast to typical angiosperm genomes, repeat (Ty3: dark blue, Ty1/Copia: medium blue, other EDTA‐annotated repeat: light blue) and gene (orange) density are remarkably consistent without an obvious repeat‐dense pericentromere surrounding putative centromeres. Gene, repeat, and male‐specific kmer (dark red) contributions were calculated within 100‐kb overlapping 2.5‐Mb sliding windows. (b) This structure is further illustrated in Chromosome 8 where the sex‐determining region (SDR) occurs, as indicated by a peak of male‐specific kmers (dark red peak on HAP2) and a conspicuous presence–absence variant. The sliding window method follows that of (a) except with smaller 250 kb widths. (c) Further zooming in on the SDR (red box) and a 500‐kb buffer reveals that the sequence insertion into the X (HAP1) sequence occurs directly in the center of the SDR and is bounded by a partially degraded 5′ sequence triplication and a 3′ large inversion/duplication. Points in the dotplot are 50‐bp overlapping 200‐bp windows of HAP1 mapped to the HAP2 sequence. Mapq is the minimap2‐defined mapping quality of these hits. Sliding windows, synteny maps and dotplots were calculated and plotted with DEEPSPACE (github.com/jtlovell/DEEPSPACE) and only used for visualization.

Despite the high degree of nucleotide and structural variation between homologous chromosomes, gene and repeat density is remarkably uniform across the *M. flabellifolia* genome. We annotated every 10‐Mb genomic window (5‐Mb overlapping) of HAP1 with 0.5–1.5 Mb (5–15%) of genic sequence and 60–82% of EDTA‐defined repeat sequences. Visually, this consistency appears to be an outlier among seed plants and more in line with examples from bryophytes (Healey *et al*., 2023). Across 1000 nonoverlapping windows, the percent of gene density SD (HAP1 = 3.6%, HAP2 = 3.8%) in both *M. flabellifolia* was closer to that of *Sphagnum fallax* (7.3%) than the highly variable eudicot genome structures of *A. thaliana* (23.0%), *M. guttatus* (21.7%), or cotton (10.6%), all of which retain the majority of their genes in repeat‐poor chromosome arms, and have few genes in the highly repetitive pericentromeric regions that surround a readily identifiable tandem‐repeat filled centromere. However, no such structural variation was apparent in *M. flabellifolia* (Fig. 2a). Not only were we unable to identify pericentromeric repetitive regions, but the centromere‐finding software TRASH was unable to identify clusters of putative centromeric repeats. When combined with a remarkably consistent 3D chromatin architecture visible in genome‐wide Hi‐C data (Fig. S1), these lines of evidence are suggestive of a holocentric chromosome organization although atypical monocentric architectures or other centromere organizations remain possible explanations. Cytogenetic and Chromatin Immunoprecipitation–based validation of holocentric chromosomes will be required to fully test this hypothesis.

### Sexual dimorphisms and identification of the sex‐determining region

Both species in *Myrothamnus* are dioecious with distinct male and female anatomy, as are some members of *Gunnera*, suggesting there could be a shared origin of dioecy before the divergence of these genera. However, the sex chromosome system in these species is not well‐understood, nor is its contribution to sexually dimorphic traits. In *M. flabellifolia*, inflorescences of both males and females consist of densely packed florets in a simple arrangement with extremely short pedicles, but male inflorescences have bracts and female inflorescences are bract‐less (Fig. 1). The male flowers develop three to six stamens that dehisce longitudinally, while females develop three basally attached carpels. Floral organs are desiccation‐tolerant in both sexes, but male flowers become desiccation‐sensitive after pollen dehiscence (Moore *et al*., 2007).

We used Illumina DNA sequencing data of 10 accessions from across geographically distinct collection sites in Namibia and South Africa (Fig. 1a) to predict the sex determination system. To test whether females or males contain the heterogametic sex chromosome pair in *M. flabellifolia*, we used a *k*‐mer‐based analysis (Carey *et al*., 2024a,b). We found four times more male‐specific than female‐specific *k*‐mers, indicating an XY sex determination system. To identify the Y chromosome in the genome assembly, and delimit the boundary of the sex‐determining region (SDR) from the recombining pseudoautosomal region (PAR), we mapped the putative Y‐specific *k*‐mers to both haplotypes. We found the SDR on the Y chromosome to be located on Chromosome 8 of HAP2 spanning only *c*. 700 kb (Fig. 2), making it among one of the smaller SDRs identified in plants, similar in size to some poplars (Renner & Müller, 2021). Despite the smaller size, there are clear structural variants between the SDR on the Y chromosome and the homologous region on the X (Fig. 2). Interestingly, the Y‐SDR is smaller than the homologous region on the X due to an X‐specific tandem triplication and accumulation of repetitive elements comprising nearly 750 kb. Whereas the Y‐specific SDR contains just six gene models, we identified 11 X‐linked homologs comparing the two haplotypes in an orthofinder v.2.5.2 analysis (Emms & Kelly, 2015, 2019). One single copy gene on the Y, with four orthologous copies within the 5' triplicated region on the X (Fig. 2) was predicted to encode a Kinesin‐1‐like protein. A homolog in rice has been shown to be important for anther dehiscence and male meiosis (Zhou *et al*., 2011), although the Arabidopsis homolog, *AtKin‐1*, influences female gametophyte development (Wang *et al*., 2014). While intriguing, functional validation of the *M. flabellifolia Kin‐1* homologs is needed to determine whether it plays a role in sex determination. The identification of the XY chromosomes, and the gene content in the SDR and homologous X‐region, provides useful information for investigating and predicting the genetic control of dioecy across the flowering plant phylogeny (Carey *et al*., 2021). For example, future work could test for a role for *Kin‐1* homologs in sex determination in *M. flabellifolia* and other dioecious plants.

The relationship between sex and desiccation tolerance in *M. flabellifolia* is unclear. No sexual dimorphisms in desiccation tolerance have been described, but our population‐level analyses identified male‐biased sex ratios in more arid environments (including the focal site) compared to female dominance in mesic regions (Marks *et al*., 2022). These patterns could be driven by dimorphic resource allocation, with females requiring more carbon and water for seed maturation and males allocating nitrogen and other resources for inflorescence and pollen production. We have also identified secondary sexual dimorphisms, including faster growth in females and greater inflorescence production in males that may influence population dynamics across environmental gradients (Marks *et al*., 2022), but do not link directly to desiccation tolerance.

### Dynamic responses to desiccation and rehydration

**Fig. 3:**
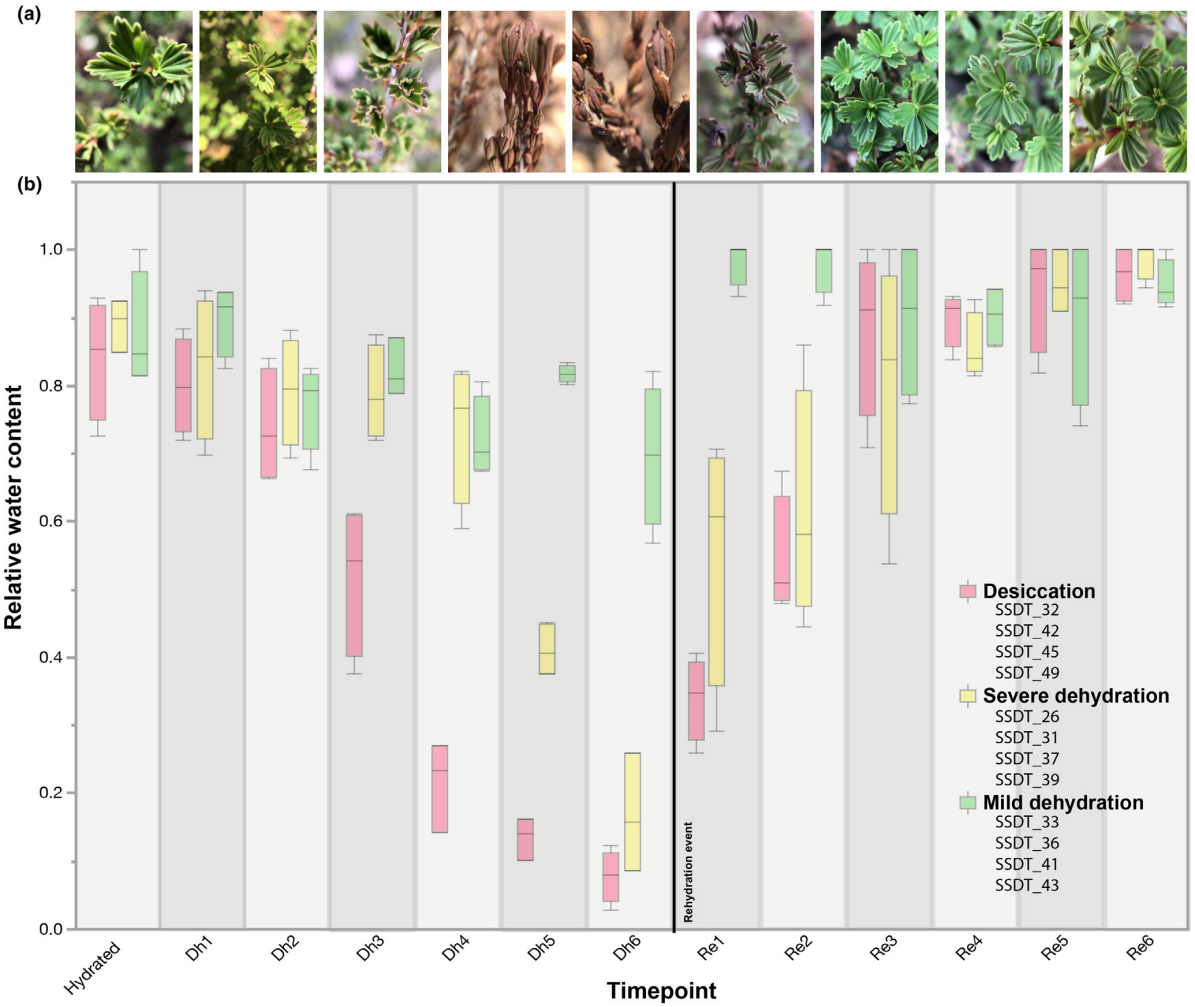
Drying dynamics of *Myrothamnus flabellifolia*. (a) Representative photographs of plants at different stages of dehydration and rehydration. (b) Relative water content (RWC) of plants throughout the 6‐d dehydration–rehydration time course. Boxplots show the median, 1^st^, and 3^rd^ quartiles, with the whiskers indicating the maximum and minimum values. Plants were sampled from a single *c*. 300 × 100 m patch at Swebe Swebe and experienced the same rainfall event (*c*. 10 mm on 18 January 2020), although individuals dried at different rates depending on local topography, soil depth, and canopy cover. Plant genotypes (e.g. SSDT_26) are listed and grouped/colored by their drying groups (‘mild dehydration,’ ‘severe dehydration,’ or ‘desiccation’) based on minimum RWC.

To better understand the molecular mechanisms of desiccation tolerance in *M. flabellifolia*, we performed high‐resolution transcriptomic profiling of 12 individual *M. flabellifolia* plants across a six‐day natural dehydration–rehydration time course in the field at Swebe Swebe Nature Reserve in Limpopo, South Africa. Plants exhibited progressive drying across 4 d without rainfall, after which a rainfall event delivered *c*. 20 mm of rain over 4 h and plants regained full hydration within 12–24 h of the rainfall event (Figs 3, S2). Despite being located within relatively close proximity (within a *c*. 300 × 100 m^2^ patch), individual plants exhibited variable drying rates and water loss, likely due to differences in microniche. We therefore classified them into three groups based on the minimum RWC they reached during the time course. Some plants desiccated rapidly and completely, with RWC dropping below 10%, and we classified these as the ‘desiccation’ group. Others experienced slower but still severe dehydration, reaching 10–30% RWC, and were classified as ‘severe dehydration’. A third group underwent only mild dehydration, with RWC declining to 60–85% and were classified as ‘mild dehydration’ (Figs 3, S2). This stratification corresponds conveniently to the early and late stages of dehydration proposed to characterize drying in resurrection plants (Farrant & Hilhorst, 2021). These drying groups were leveraged for statistical comparisons between mild dehydration, severe dehydration, and full desiccation.

#### Overview of broad patterns via dimension reduction analyses

**Fig. 4:**
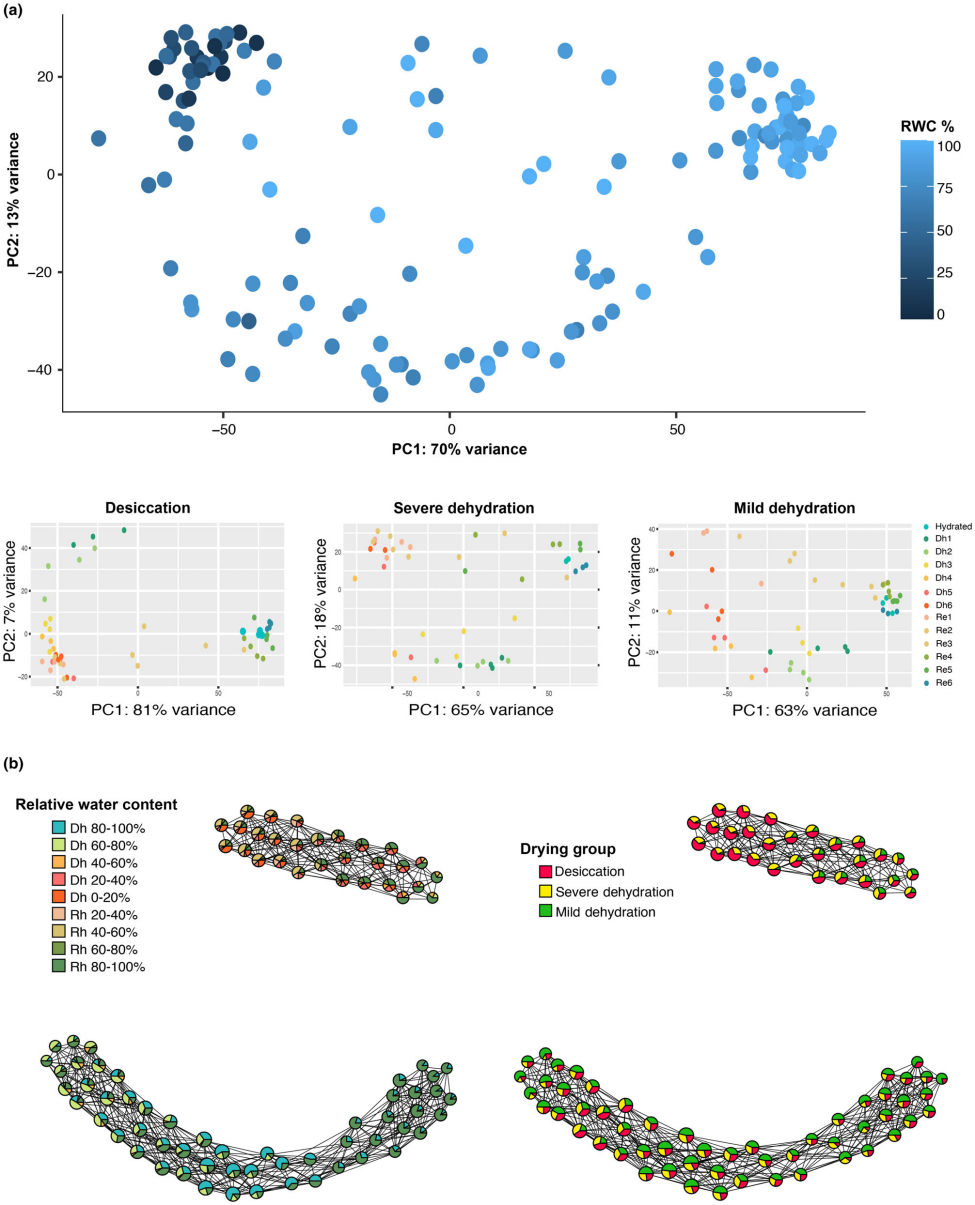
Gene expression of *Myrothamnus flabellifolia* during the dehydration time course. (a) Principal component analysis (PCA) of transcript abundance for all plants colored by relative water content. Additional PCAs show transcript abundance for each of the different drying groups, colored by sampling time. Across analyses, the strongest separation of samples corresponds to hydration status rather than genotype or sex (Supporting Information Fig. S3). (b) Topological data analyses of transcript abundance. This network‐based approach captures nonlinear relationships and global structure in the data beyond what PCA alone can resolve. Each node on the graph represents a cluster of similar RNA‐seq samples with similar expression profiles and edges indicating shared samples between clusters. The node color depicts the identity of samples within that cluster, shown here by both RWC (left panel) and Drying Group (right panel).

To visualize broad changes in transcript abundance across the dehydration–rehydration time course, we performed PCA. PCA revealed a strong association between transcript abundance and RWC, with the first principal component (PC1) explaining 70% of the total variation in transcript abundance and separating samples primarily on water status (Fig. 4a). There was no notable pattern related to the sex or genotype of samples (Fig. S3). We also conducted targeted PCA for each drying group separately, which revealed increasing segregation of samples under more severe dehydration, highlighting an escalating transcriptomic response to progressive dehydration (Fig. 4a).

Although PCA provided some dimensionality reduction, residual variability–arising from heterogeneity, experimental noise, or genotype‐level differences in the data–may have masked underlying biological patterns. To address this, we applied TDA using the Mapper algorithm (van Veen *et al*., 2019; Palande *et al*., 2023; Marks *et al*., 2024), a network‐based approach that is capable of capturing the shape of high‐dimensional, sparse datasets. This approach is particularly well‐suited for transcriptomic datasets, because it can reveal nonlinear structure and trajectories that PCA often compresses into residual variance. Mapper requires a lens function, a user‐defined feature that shapes how the data are clustered and connected. We used RWC as the lens, anchoring it to the fully hydrated condition, allowing us to explore expression changes along a continuum of dehydration and recovery. The resulting Mapper graph shows that water status was the primary driver of expression variation, followed by the imposed drying group classification (Fig. 4b). Again, we found no clear patterns related to the sex or genotype of accessions, indicating that transcriptomic changes during desiccation and rehydration are predicted primarily by water status and drying severity, with minimal differences across individuals, genotypes, or sexes.

#### Differentially abundant transcripts showcase an escalating response to desiccation

**Fig. 5:**
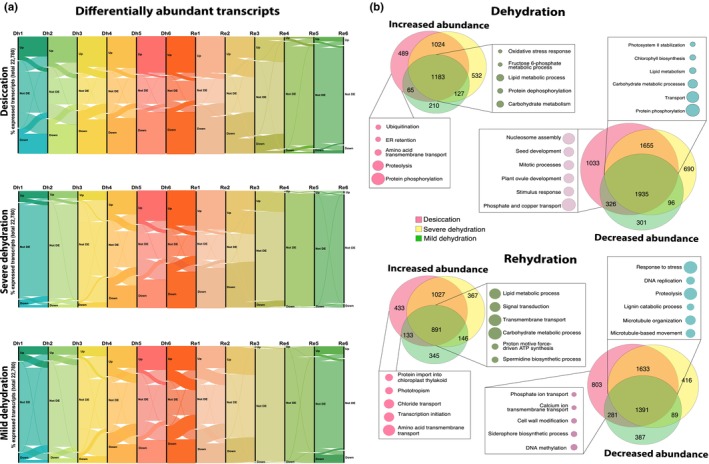
Differentially abundant transcripts in *Myrothamnus flabellifolia*. (a) Alluvial diagrams showing the number and flow of differentially abundant transcripts at each time point for each of the three drying groups. All 22 780 expressed transcripts are included in the plots and the proportion ‘Up’, ‘Down’, and ‘Not DE’ transcripts are shown. These plots highlight the balance between up‐ and downregulated genes and an increase in the total number of differentially abundant transcripts (DATs) as dehydration severity intensifies. (b) Venn diagrams showing the number of overlapping and unique DATs across the three drying groups. These diagrams illustrate both the large core set of transcripts consistently differentially expressed across all groups, as well as subsets unique to the most severe dehydration A selection of the prominent enriched gene ontology categories is shown for the shared set (highlighting general stress and metabolic processes) and for the unique subsets (highlighting pathways activated specifically under extreme desiccation). The size of the circles for Gene Ontology terms is proportional to the number of significant genes within that term.

Next, we quantified changes in transcript abundance throughout the dehydration–rehydration time course by identifying DATs using the thresholds of Log_2_FC > |2| and Bonferroni‐adjusted *P*‐values < 0.05. DATs were identified separately for each drying group by comparing transcript abundance at each sampling time point to baseline hydrated conditions. This approach allowed us to differentiate between responses associated with the severity of water loss. Our results revealed an escalation in the number of DATs as dehydration intensified throughout the time course (Fig. 5a). Upon rehydration, transcript abundance largely returned to baseline levels, highlighting the reversible nature of these molecular changes (Fig. 5a). Unsurprisingly, changes in transcript abundance were more pronounced in plants that underwent full desiccation compared to those that only experienced mild dehydration (Fig. 5a). In plants that underwent full desiccation, > 60% of the 22 780 expressed transcripts exhibited significant changes in abundance, but even under mild dehydration > 40% of the expressed transcripts exhibited differential abundance, reflecting the early induction and massive transcriptomic remodeling associated with drying.

To capture the key transcriptomic shifts in each drying group, we generated nonredundant lists of DATs that either increased or decreased in abundance during dehydration, and separately during rehydration. Comparing these lists across the three drying groups revealed both shared and unique changes in transcript abundance (Fig. 5b). Most DATs were common to all drying groups, reflecting an early activation of water‐deficit responses initiated by all plants. However, some unique DATs were identified only during the later stages of desiccation. These DATs likely represent responses to, and consequences of, the intense stress of full desiccation, and provide valuable insights into the progressive activation and repression of molecular pathways during desiccation and rehydration.

We conducted GO enrichment analysis of shared and unique DATs to identify key pathways impacted by dehydration and rehydration (Fig. 5b). During dehydration, all plants exhibited the classic decrease of photosynthesis‐related processes, including Chl biosynthesis, coupled with reductions in fatty acid biosynthesis, lipid metabolism, DNA replication, and cell wall modification (e.g. xyloglucan metabolism). These changes likely reflect an overall energy‐saving strategy to minimize metabolic demands and damage due to water stress. Even in plants experiencing just mild dehydration, auxin responses and glutamine biosynthesis were suppressed, suggesting an early shift away from growth to prioritize survival under water‐deficit conditions. Plants that experienced full desiccation displayed unique reductions in cell division, mitotic processes, ovule development, stimulus response, and phosphate and copper transport, indicating a more complete halt in growth and resource redistribution (Fig. 5b).

As expected, pathways related to oxidative and salt stress increased in all plants during drying. Even under mild drought plants exhibited an increase in transcripts related to carbohydrate metabolism (e.g. sucrose transport, glycolysis, glyoxylate cycle, and malate metabolism), reflecting the well‐described role of carbohydrate metabolism in enhancing osmoprotection, vitrification, and cellular defense during desiccation. During severe dehydration, transcripts related to spermidine and spermine biosynthesis, vitamin E, and terpenoid biosynthesis were enriched, which are likely involved in cellular protection against oxidative damage. RNA regulation, nucleotide biosynthesis, and RNA surveillance were also abundant during more extreme dehydration indicating changes in nucleic acid regulation. During the most severe stages of desiccation, protein phosphorylation, ubiquitination, ER retention, and proteolysis pathways were enriched, suggesting that mechanisms to manage protein stability and recycle damaged proteins are critical during the final stages of desiccation (Fig. 5b).

During rehydration, DNA replication, microtubule organization, and cell wall‐related processes (e.g. cellulose and microfibril organization) remained suppressed. Processes related to cell growth, phototropism, and flowering also showed persistent suppression, suggesting a prolonged delay in growth and focus on repair and recovery before regeneration. Plants that underwent full desiccation showed a unique reduction in DNA methylation and phosphate and calcium transport, during rehydration, possibly reflecting long‐term expression remodeling via epigenetic marks (Fig. 5b).

Many transcripts increased during rehydration in all plants, including those related to spermine and spermidine biosynthesis, ethanol oxidation, sulfate assimilation, terpenoid biosynthesis, and RNA surveillance, suggesting a balance between continued stress response and metabolic recovery. In plants that experienced full desiccation, autophagy and ribosomal subunit biogenesis were uniquely upregulated pointing toward extensive repair mechanisms needed to restore normal cellular function after desiccation (Fig. 5b). Taken together, these patterns highlight the interplay between preemptive suppression of growth and activation of protective mechanisms during dehydration, coupled with rehydration‐activated repair mechanisms required for recovery. The ability to halt growth while upregulating defense and repair pathways is likely critical to survival and recovery from desiccation.

#### Modules of co‐expressed genes exhibit dynamic activation and repression

**Fig. 6:**
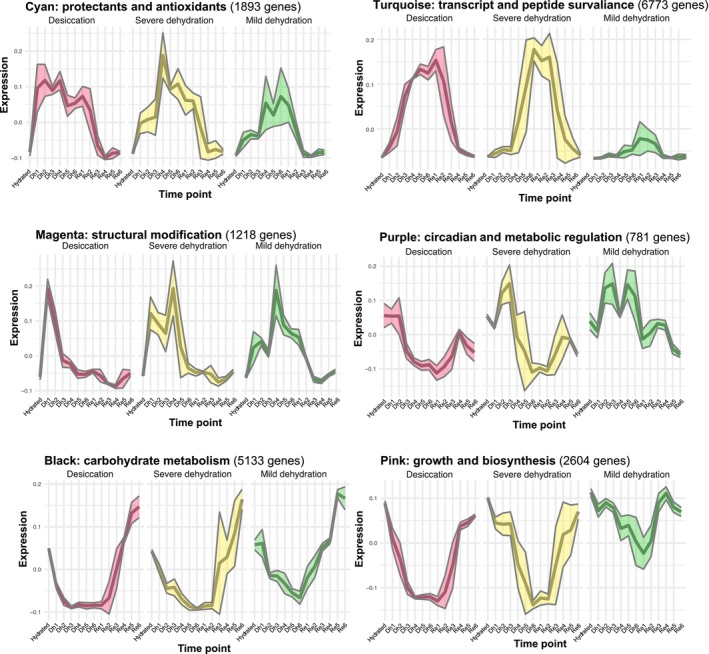
Expression profiles of co‐expressed modules in each of the three drying groups of *Myrothamnus flabellifolia*. Modules of co‐expressed genes are shown for each of the three drying groups, with the left column highlighting modules that display similar expression in all drying groups, and the right column showing modules contrasting expression across different drying groups. We include broad functional labels for each module based on Gene Ontology enrichment results.

To further explore shifts in transcript abundance across the dehydration–rehydration time course, we identified modules of co‐expressed genes and plotted the expression of these modules across the time course for each of the three drying groups (Fig. 6). Many of the modules exhibited similar expression patterns in all of the drying groups (e.g. black, cyan, and magenta modules; Fig. 6, left‐hand column) and these likely represent core responses to water loss established in the early stages of drying. These modules were enriched for GO terms involved in carbohydrate‐related processes such as gluconeogenesis, glycolysis, TCA cycle, sucrose, trehalose, glucose, and fructose‐6‐phosphate metabolism, as well as cell wall biogenesis and modification paralleling the DAT results, which also identified shifts in carbohydrate‐related processes and cell wall modifications early in dehydration. These shared modules contained other hallmarks of desiccation tolerance, such as superoxide metabolism, glutathione and glutamine biosynthesis, and Gamma‐Aminobutyric Acid (GABA) catabolism, indicating that protective mechanisms against oxidative damage and metabolic adjustments are also initiated early in the dehydration process.

In contrast to the broadly conserved modules, certain key modules (e.g. pink, purple, and turquoise modules; Fig. 6, right‐hand column) displayed distinct expression patterns related to the severity of dehydration. The pink module, for example, was downregulated only in plants experiencing severe dehydration and desiccation but remained near baseline levels under mild dehydration. This module was functionally enriched for nucleotide biosynthesis, cytoskeleton‐related processes, and amino acid‐related pathways, suggesting that processes related to cell division and growth are increasingly downregulated as dehydration severity increases paralleling the DAT analysis described previously. By contrast, the turquoise module was only activated in severe dehydration and desiccation and was enriched for GO terms associated with nucleic acid regulation, including telomere maintenance, chromatin remodeling, DNA‐templated transcription, mRNA splicing, and amino‐tRNA aminoacylation, suggesting increasing surveillance of transcription and translation in later stages of drying. The turquoise module also included terms related to protein homeostasis, such as proteolysis and protein trafficking, similar to the DAT analysis that pointed to an increase in protein phosphorylation and ubiquitination during more extreme stages of dehydration. The purple module was uniquely activated in plants experiencing mild dehydration but suppressed in plants experiencing more extreme dehydration and desiccation. This module was enriched for circadian regulation, phototropism, fatty acid beta‐oxidation, protein secretion, and spliceosomal snRNP assembly, suggesting that early‐stage responses may be aimed at maintaining homeostasis until some threshold is crossed. The downregulation of these processes in more extreme conditions indicates a shift away from maintaining normal cellular function toward a committed prioritization of survival mechanisms. Together, these findings suggest that plants experiencing severe dehydration activate specialized pathways for genomic stability, transcriptional regulation, and protein quality control that may be key for survival under extreme water stress.

#### Antioxidant capacity and implications for medicine

The resilience of *M. flabellifolia* to desiccation can be partly attributed to its rich composition of phenolic compounds (Bentley *et al*., 2019), which play an important role in protecting the plant against oxidative stress and have intriguing medicinal properties. Flavonoids, hydroxycinnamic acids, and anthocyanins, among others, contribute significantly to the antioxidant capacity of *M. flabellifolia* by acting as free‐radical scavengers targeting ROS, such as superoxide radicals, which are generated during periods of stress. These phenolic compounds help maintain the structural integrity of plant tissues and protect cells from oxidative damage (Kranner *et al*., 2002; Moore *et al*., 2005). The same antioxidant and stress‐mitigating properties that confer desiccation tolerance are also key to the medicinal relevance of *M. flabellifolia*, as many of these compounds exhibit pharmacological potential, including anti‐inflammatory and neuroprotective effects.

Several GO terms linked to radical scavenging activity and stress responses were identified across the co‐expression modules activated during dehydration. In the turquoise co‐expression module, which peaked most dramatically under both desiccation and severe dehydration (Fig. 6), several GO terms were closely linked to redox homeostasis (e.g. superoxide metabolic process and glutathione biosynthetic process), metabolic pathways (e.g. TCA cycle and glycolytic process), and signaling pathways (e.g. intracellular signal transduction). The abundance of phenolic compounds in desiccated *M. flabellifolia* tissues (Bentley *et al*., 2019) aligns with these transcriptomic responses. Specifically, gallic acid and its galloyl derivatives, ellagic acid derivatives, various quercetin derivatives, and anthocyanins (such as delphinidin and cyanidin glycosides) are strongly associated with superoxide metabolic processes, while naringenin, quercetin derivatives, and anthocyanins modulate intracellular signal transduction pathways, thereby influencing cellular stress responses and potentially contributing to their medicinal efficacy. These functions, while crucial for desiccation tolerance, may also be relevant for biomedical applications, particularly in understanding how antioxidant compounds can contribute to genome stability and cellular repair mechanisms in other biological systems.

#### Dynamic changes in the abundance of key genes highlight complex regulatory networks

Both the LEA and ELIP gene families have well‐documented roles in desiccation tolerance (VanBuren *et al*., 2019; Hernández‐Sánchez *et al*., 2022), and we conducted targeted analyses to examine changes in the abundance of LEA and ELIP transcripts in *M. flabellifolia* across the dehydration–rehydration time course. LEAs have long been recognized as key players in stress response and are generally regarded as molecular protectants thought to stabilize proteins and membranes during water loss. LEAs typically have a high degree of intrinsic disorder (Hernández‐Sánchez *et al*., 2022) and have roles in stabilizing the bioglasses generated during desiccation (Rascio & Rocca, 2005). ELIPs, on the other hand, have emerged more recently as critical players in desiccation tolerance, with a presumed role in preventing photooxidative damage through their Chl‐binding activities (Hutin *et al*., 2003). ELIPs are significantly expanded in the genomes of all sequenced resurrection plants and typically occur in tandem arrays (VanBuren *et al*., 2019; Marks *et al*., 2024).

**Fig. 7:**
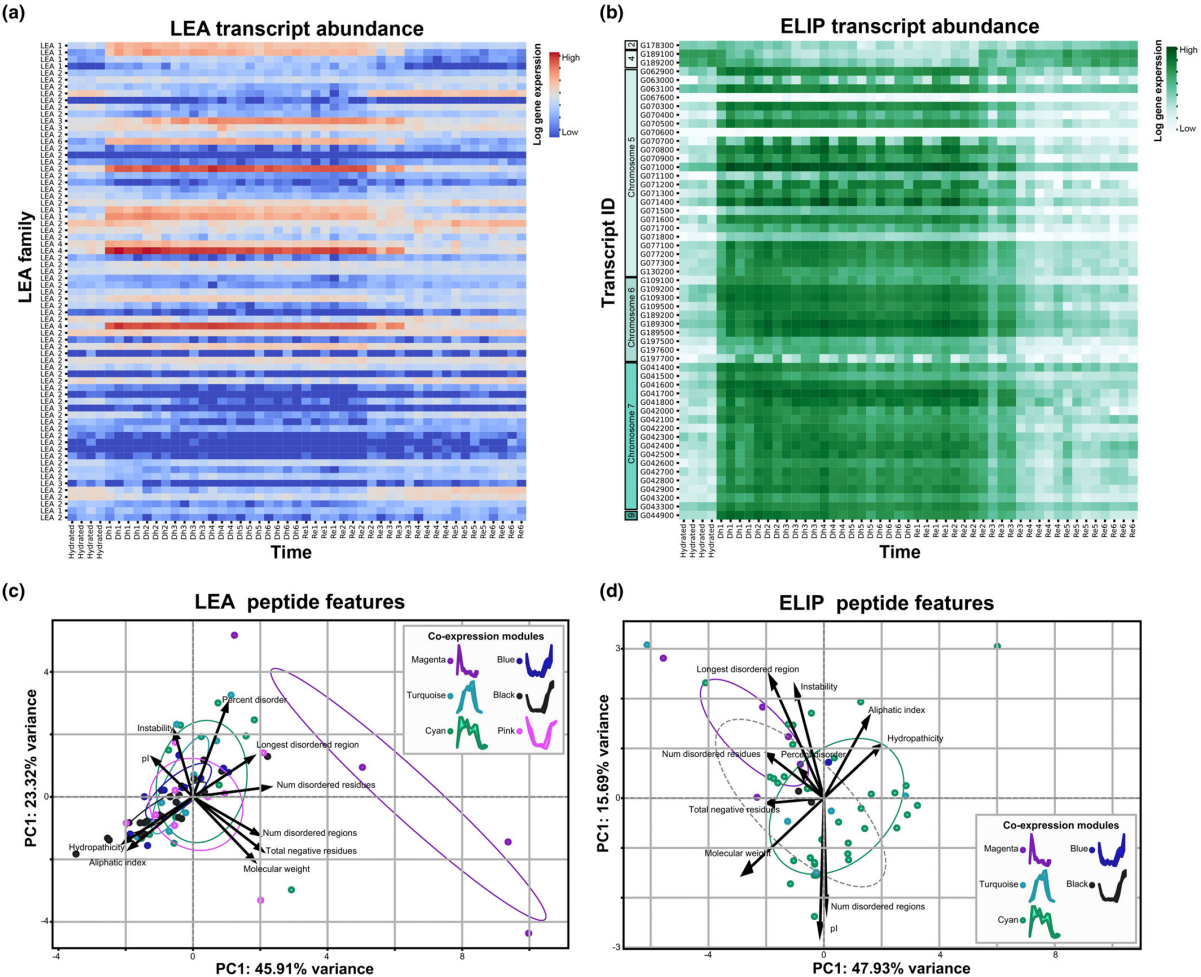
Expression dynamics of late embryogenesis abundant (LEA) and early light‐induced proteins (ELIP) transcripts in *Myrothamnus flabellifolia*. (a) Heatmap of log‐transformed transcript abundance levels of LEA transcripts across the dehydration–rehydration time course (*x*‐axis). LEAs are grouped and ordered by family (e.g. LEA1, LEA2, LEA6) to highlight patterns related to their known structural and functional features. (b) Heatmap of ELIPs across the time course. ELIPs are ordered by their genomic positions and labeled by gene ID to highlight their tandem array organization and genomic clustering. Data are only shown for plants that experienced full desiccation. Principal component analysis of predicted peptide characteristics (intrinsic disorder, hydropathicity, molecular weight, and charge) colored by co‐expression module membership for (c) LEAs and (d) ELIPs.

Both LEAs and ELIPs are among the most highly expressed transcripts during desiccation across numerous species, and *M. flabellifolia* is no exception. Many *M. flabellifolia* LEA and most ELIP transcripts increased dramatically in abundance during dehydration. Interestingly, a few specific LEA and ELIP transcripts showed the opposite pattern – with elevated abundance during hydrated conditions that declined as dehydration progressed. This pattern could indicate either a baseline level of protection via constitutive expression, a ‘priming’ response from prior desiccation events, or a role for these transcripts in other unrelated processes. As dehydration progressed, expression shifted from one set of LEAs and ELIPs to a different subset, indicating a possible transition from general protective mechanisms to those optimized specifically for dehydration and desiccation (Fig. 7a,b).

To better understand the nature of these dynamic changes, we investigated differences in the disorder, hydropathicity, molecular weight, and charge of each predicted LEA and ELIP peptide and tested whether any of these peptide properties were related to their expression profiles. In general, LEAs with higher hydropathic indices (more hydrophobic) were less disordered (*r* = −0.65), suggesting a trade‐off between hydrophobicity and flexibility (Fig. 7c). More importantly, we found that the more disordered LEA proteins were expressed predominantly during desiccation (i.e. membership in magenta, cyan, turquoise co‐expression modules), while less disordered LEAs exhibited reduced expression during this phase (i.e. blue, black, and pink modules). These trends were closely associated with LEA family classification, with LEA2 transcripts being less disordered and expressed at lower levels during dehydration compared with the highly disordered and more abundant LEA1 and LEA6 transcripts. We hypothesize that more disordered LEA transcripts may act as flexible molecular chaperones, stabilizing a broad array of cellular targets during desiccation, while less disordered variants may provide other functions. This link between transcript abundance and structural diversity of LEA proteins suggests specialized roles in stress tolerance across different physiological contexts.

Our analyses revealed intriguing patterns within the ELIPs as well. Similar to other resurrection plants (VanBuren *et al*., 2019), ELIP genes are expanded in tandem arrays in *M. flabellifolia* (Fig. 7b). The majority of *M. flabellifolia* ELIPs exhibit the typical dehydration‐induced increases in abundance (Fig. 7b) and were clustered in the cyan co‐expression module, suggesting a coordinated role in desiccation response (Fig. 7d). However, a small number of these ELIPs had little to no expression under any condition and could be pseudogenes, and a few other ELIPs were expressed only under hydrated conditions – especially those located in the small array on Chromosome 4 (Fig. 7b). Analysis of the peptide characteristics confirmed that ELIPs generally exhibit lower levels of intrinsic disorder compared with LEAs. Consequently, their expression patterns show weaker associations with peptide properties. However, higher disorder in ELIPs appeared to be weakly linked with earlier induction during drying.

To better understand the drivers of both ELIP and LEA expressions, we investigated the CREs associated with each gene using the PLACE v.30.0 database (Higo *et al*., 1999). We tested for enrichment of CREs in LEAs and ELIPs that were upregulated during dehydration (i.e. magenta, turquoise, and cyan modules) vs those that were downregulated during dehydration (i.e. blue, black, and pink modules).

Dehydration‐induced LEAs were significantly enriched for multiple stress‐related CRE motifs, including WRKY‐ and abscisic acid‐responsive elements, as well as light‐associated motifs, consistent with the activation of conserved drought‐responsive pathways. By contrast, the single CRE enriched for downregulated LEAs was a metal/oxygen‐responsive G‐box family element, suggesting possible functions outside of desiccation tolerance (Table 2).

**Table 2:** Enriched *cis*‐regulatory elements of *Myrothamnus flabellifolia* associated with up‐ and downregulated late embryogenesis abundant and early light‐induced protein (ELIPs) during dehydration, identified using the PLACE v.30.0 database.

Gene family	Expression pattern	Enriched CRE(s)	TF and putative functions	Citation	*P*‐value
LEA	Upregulated	WBOXNTCHN48	WRKY TFs (abiotic stress)	Bakshi & Oelmüller (2014); Xiang *et al*. (2021); Yang *et al*. (2023)	0.0097
LEA	Upregulated	CCA1ATLHCB1	CCA1 (light‐harvesting regulation)	Wang *et al*. (1997); Wang & Tobin (1998); Sun *et al*. (2019)	0.0302
LEA	Upregulated	IRO2OS	OsIRO2 (iron uptake/homeostasis)	Ogo *et al*. (2006); Li *et al*. (2022)	0.0204
LEA	Upregulated	BOXIIPCCHS	G‐box‐like (light regulation)	Block *et al*. (1990)	0.0273
LEA	Upregulated	ACGTABREMOTIFA2OSEM	ABRE (Abscisic acid (ABA)‐responsive element)	Narusaka *et al*. (2003); Zhang *et al*. (2024)	0.0245
LEA	Upregulated	MYB1LEPR	R2R3‐MYB (ABA/drought)	Abe *et al*. (1997); Chakravarthy *et al*. (2003); Giarola *et al*. (2017)	0.0371
LEA	Downregulated	CURECORECR	Metal/oxygen responses; G‐box family	Quinn & Merchant (1995); Sharma *et al*. (2011)	0.0232
ELIP	Upregulated (trend)	MYCCONSENSUSAT	MYC TFs (ABA, dehydration)	Abe *et al*. (2003)	0.0955
ELIP	Upregulated (trend)	TAAAGSTKST1	Guard cell function	Plesch *et al*. (2001)	0.0592
ELIP	Downregulated	TATCCACHVAL21	GA‐responsive motif (germination)	Gubler & Jacobsen (1992); Isabel‐LaMoneda *et al*. (2003); Martínez *et al*. (2005)	0.0443
ELIP	Downregulated (trend)	TATCCAYMOTIFOSRAMY3D	GA‐responsive element	Xie *et al*. (2016)	0.0955

Only motifs with *P* < 0.05 are considered significant while motifs with *P* < 0.1 are reported as trends. Transcription factor associations and functions are provided based on published studies with references included in the Citation column.

ELIPs showed weaker CRE enrichment overall. The only significantly enriched motif occurring in downregulated ELIPs was a GA‐responsive element, consistent with canonical roles of ELIPs in germination and early seedling development. Additional motifs showed trends toward enrichment (*P* < 0.1), including another GA‐responsive element in downregulated ELIPs, compared to stress‐associated motifs in the CREs of upregulated ELIPs, suggesting possible regulatory divergence and neofunctionalization (Table 2).

## Discussion

The chromosome‐level, haplotype‐resolved genome assembly of *M. flabellifolia* presented here provides a foundational resource for understanding the genomic architecture and resilience of this iconic resurrection plant. The *M. flabellifolia* genome exhibits strikingly uniform gene and repeat density across chromosomes and lacks clear evidence of conventional centromeres or pericentromeric regions. These features point toward an unusual and potentially adaptive genomic configuration that warrants further cytogenetic investigation. The two haplotypes are highly divergent, exhibiting substantial structural variation, including a large inversion on Chromosome 1 and widespread gene presence–absence and copy number variation, which may contribute to functional diversity in this dioecious outcrossing species.

We identified and characterized an XY sex determination system in *M. flabellifolia*, with heterogametic males and a small, *c*. 700 kb SDR located on Chromosome 8. The SDR contains just six gene models, including a kinesin‐like gene previously implicated in anther development in rice (Zhou *et al*., 2011), along with two Y‐specific genes of unknown function. This compact and minimally differentiated SDR offers a rare opportunity to study the evolution of dioecy and sexual dimorphism in a nonmodel lineage under extreme environmental stress. Although no direct differences in desiccation tolerance between sexes have been reported, population‐level studies reveal skewed sex ratios along environmental gradients, with males more common in arid environments and females more frequent in mesic areas (Marks *et al*., 2022).

We profiled 12 plants during a natural dehydration–rehydration event in the field, making our study one of the first to survey an angiosperm resurrection plant directly in the field. In general, transcriptomic changes were tightly linked to changes in RWC, reflecting the central role of water availability in altering cellular processes. Early transcriptomic changes, such as the general suppression of photosynthesis and growth, parallel previous findings and point toward an energy‐saving strategy that helps to minimize oxidative damage (Farrant, 2000; Pardo *et al*., 2020; Farrant & Hilhorst, 2021; Marks *et al*., 2023; VanBuren *et al*., 2023). As desiccation progressed, a transition to more specialized responses occurred, including the suppression of cell division and circadian rhythm, and a shift toward a more complete cessation of cellular activity coupled with mechanisms to preserve molecular integrity. These findings generally align with the early and late stages of dehydration proposed for desiccation‐tolerant taxa, which are characterized by the early suppression of metabolic activity to conserve energy and minimize damage during desiccation (Farrant, 2000; Alejo‐Jacuinde *et al*., 2020; Oliver *et al*., 2020). Co‐expression analysis further highlighted the complexity of desiccation responses in *M. flabellifolia* and reinforced the evidence for an escalating response as cellular dehydration increased. Modules enriched for GO terms related to maintaining cellular stability and energy production during drought such as carbohydrate metabolism, oxidative stress responses, and cell wall modifications were activated early during the dehydration process in all plants. These modules included many of the known hallmarks of desiccation tolerance, such as trehalose and raffinose biosynthesis (Dace *et al*., 2023), pointing toward the early activation of key protective strategies. However, modules with severity‐specific activation indicate that mechanisms of transcription and translation regulation are induced at later stages, and point toward an escalating response that is programmed to match the intensity of dehydration stress.

Changes in LEA and ELIP expressions during dehydration and rehydration reinforce their central role in protecting cells under extreme dehydration (VanBuren *et al*., 2019; Kc *et al*., 2024). Elevated expression of specific LEA and ELIP transcripts in hydrated plants suggests either a baseline level of protection via constitutive expression, a primed state driven by past desiccation events that may equip *M. flabellifolia* for rapid and unexpected water loss events that are frequent in its native habitat, or unrelated roles in other processes. However, further experimental work would be needed to test these hypotheses. During dehydration, expression shifted to increasingly disordered sets of LEA and ELIP genes, pointing toward a transition of mechanisms and highlighting the subtle but important role of intrinsic disorder in providing protection during desiccation.

The significant enrichment of WRKY‐binding W‐box motifs, ABA‐responsive elements, and light‐regulatory CREs in upregulated LEA genes suggests that dehydration‐induced expression of these genes is mediated through well‐established drought‐responsive pathways (Abe *et al*., 1997; Xiang *et al*., 2021), and that light may also play a role in modulating LEA gene expression under dehydration stress. Interestingly, only one significant CRE was identified in downregulated LEA genes, implying that dehydration responses in *M. flabellifolia* primarily involve induction rather than repression of LEA genes. This activation‐based regulation aligns with the physiological role of LEA proteins, in which their accumulation is thought to help stabilize cellular structures and prevent protein aggregation during desiccation (Kc *et al*., 2024).

CRE enrichments also point to functional divergence among *M. flabellifolia* ELIPs. The small subset of ELIP genes that were downregulated during dehydration appears to retain ancestral regulatory features (Hutin *et al*, 2003), including promoter motifs responsive to gibberellin and light, both of which are traditionally linked to seed germination and photomorphogenic development (Fleet & Sun, 2005). This pattern suggests that these downregulated ELIPs may fulfill a conserved role in early seedling establishment but are not required – or are actively repressed – during desiccation. By contrast, the majority of ELIPs are highly expressed during desiccation and lack enrichment for these classical GA motifs. Many of these upregulated ELIPs are found in large tandem arrays on Chromosomes 5, 6, and 7, consistent with a history of gene duplication and potential neofunctionalization. Several motifs associated with stress responses and protective functions trended toward enrichment in these dehydration‐induced ELIPs, suggesting the evolution of desiccation‐specific regulatory modules. Taken together, these data support a model in which ancestral ELIPs serve canonical germination‐related functions under GA and light control, while duplicated copies have acquired new regulatory sequences that allow them to function during desiccation, although functional validation is needed to confirm this hypothesis.

Taken together, this study showcases the unique genomics of *M. flabellifolia* and provides an in‐depth characterization of the complex biology of desiccation tolerance. As one of only two extant genera in the core eudicot order Gunnerales, *Myrothamnus* occupies a key phylogenetic position for comparative evolutionary studies. The ability to survive complete desiccation, coupled with a dioecious reproductive system, and the production of antioxidant‐rich secondary metabolites with known medicinal value, makes *M. flabellifolia* a powerful system for linking evolutionary genomics, stress biology, and applied plant science. This genome assembly not only enables deeper insight into the molecular mechanisms of desiccation tolerance and sex determination but also provides a foundation for future research into the ecological strategies and biotechnological potential of this iconic resurrection plant.

## Competing interests

None declared.

## Author contributions

RAM, JMF, RV and JLM conceived of the study. RAM, JT, KB and JG collected and generated the data. RAM, SBC, JTL, AH, TB, NMC, JS, AL, CMM, CP, JY, DB, JW and JWJ analyzed the data. RAM, SBC, JTL, LVDP, NMC, JB, AH, RV, JLM and JMF interpreted the data. RAM wrote the manuscript with sections contributed by SBC, JTL, TB, JB and LVDP. All authors reviewed and approved the final manuscript.

## Disclaimer

The New Phytologist Foundation remains neutral with regard to jurisdictional claims in maps and in any institutional affiliations.

## Supporting information


**Fig. S1** Hi‐C contact map of *Myrothamnus flabellifolia* showing the remarkably consistent 3D chromatin architecture visible in genome‐wide Hi‐C data.
**Fig. S2** Relative water content of *Myrothamnus flabellifolia* plants throughout the 6‐d dehydration–rehydration time course.
**Fig. S3** Principal component analysis (PCA) of *Myrothamnus flabellifolia* transcript abundance for all plants colored by sex and genotype.
**Table S1** Genomic libraries included in the *Myrothamnus flabellifolia* (*var. SSDT_37*) genome assembly.
**Table S2** PACBIO CCS library statistics for the libraries included in the *Myrothamnus flabellifolia* (*var. SSDT_37*) genome assembly.
**Table S3** Summary statistics of the initial output of the HAP1 RACON polished HiFiAsm + HIC assembly of *Myrothamnus flabellifolia*.
**Table S4** Summary statistics of the initial output of the HAP2 RACON polished HiFiAsm + HIC assembly of *Myrothamnus flabellifolia*.
**Table S5** Final summary assembly statistics for the v.1.0 HAP1 chromosome scale assembly of *Myrothamnus flabellifolia*.
**Table S6** Final summary assembly statistics for the v.1.0 HAP2 chromosome scale assembly of *Myrothamnus flabellifolia*.
Please note: Wiley is not responsible for the content or functionality of any Supporting Information supplied by the authors. Any queries (other than missing material) should be directed to the *New Phytologist* Central Office.

## Data Availability

Genome assemblies and annotations are available at NCBI under bioproject no. PRJNA1114142 and at the Department of Energy's Joint Genome Institute genome portal: https://phytozome.jgi.doe.gov/info/Mflabellifoliavar_SSDT_37HAP1_v1_1 and https://phytozome.jgi.doe.gov/info/Mflabellifoliavar_SSDT_37HAP2_v1_1 as part of the open green genomes project (https://phytozome.jgi.doe.gov/ogg). DNA sequencing reads are deposited in NCBI's SRA under the umbrella project BioProject no. PRJNA1114756. All RNA sequencing reads have been deposited in NCBI's SRA under BioProject nos. PRJNA1114143–PRJNA1114253, and accession nos. SRR29328105, SRR29328106, SRR29328107, SRR29328108, SRR29328109, SRR29328110, SRR29328111, SRR29328112, SRR29328113, SRR29328114, SRR29328115, SRR29328116, SRR29328117, SRR29328119, SRR29328120, SRR29328121, SRR29328122, SRR29328123, SRR29328124, SRR29328125, SRR29328126, SRR29328127, SRR29328128, SRR29328129, SRR29328130, SRR29328131, SRR29328132, SRR29328133, SRR29328134, SRR29328135, SRR29328136, SRR29328137, SRR29328138, SRR29328139, SRR29328140, SRR29328141, SRR29328142, SRR29328143, SRR29328144, SRR29328145, SRR29328170, SRR29328171, SRR29328172, SRR29328173, SRR29328174, SRR29328175, SRR29328176, SRR29328177, SRR29328178, SRR29328179, SRR29328180, SRR29328181, SRR29328182, SRR29328183, SRR29328199, SRR29328200, SRR29328201, SRR29328202, SRR29328203, SRR29328204, SRR29328211, SRR29328212, SRR29328213, SRR29328214, SRR29328220, SRR29328222, SRR29328223, SRR29377105, SRR29377106, SRR29377107, SRR29377108, SRR29377109, SRR29377110, SRR29377111, SRR29377112, SRR29377113, SRR29377114, SRR29377115, SRR29377116, SRR29377117.
