# Supplementary material for: The architecture of resilience: a genome assembly of Myrothamnus flabellifolia sheds light on desiccation tolerance and sex determination
Source: New Phytol. 2025 Nov 2;249(2):1063–84. doi: 10.1111/nph.70700 (PMC12712429; doi:10.1111/nph.70700)
Supplement: Supplementary file 1 — Fig. S1 Hi‐C contact map of Myrothamnus flabellifolia showing the remarkably consistent 3D chromatin architecture visible in genome‐wide Hi‐C data. Fig. S2 Relative water content of Myrothamnus flabellifolia plants throughout the 6‐d dehydration–rehydration time course. Fig. S3 Principal component analysis (PCA) of Myrothamnus flabellifolia transcript abundance for all plants colored by sex and genotype. Table S1 Genomic libraries included in the Myrothamnus flabellifolia (var. SSDT_37) genome assembly. Table S2 PACBIO CCS library statistics for the libraries included in the Myrothamnus flabellifolia (var. SSDT_37) genome assembly. Table S3 Summary statistics of the initial output of the HAP1 RACON polished HiFiAsm + HIC assembly of Myrothamnus flabellifolia. Table S4 Summary statistics of the initial output of the HAP2 RACON polished HiFiAsm + HIC assembly of Myrothamnus flabellifolia. Table S5 Final summary assembly statistics for the v.1.0 HAP1 chromosome scale assembly of Myrothamnus flabellifolia. Table S6 Final summary assembly statistics for the v.1.0 HAP2 chromosome scale assembly of Myrothamnus flabellifolia. Please note: Wiley is not responsible for the content or functionality of any Supporting Information supplied by the authors. Any queries (other than missing material) should be directed to the New Phytologist Central Office. [file NPH-249-1063-s001.pdf]

New Phytologist Supporting Information

Article Title: The architecture of resilience: a genome assembly of *Myrothamnus flabellifolia* sheds light on desiccation tolerance and sex determination

Authors: Rose A. Marks, John T. Lovell, Sarah B. Carey, Llewelyn Van Der Pas, Nyaradzai Chimukuche, Tomáš Brůna, Christopher Plott, Jenell Webber, Anna Lipzen, Juying Yan, Diane Bauer, Joanne Bentley, Jayson Talag, Chloee M. McLaughlin, Kerrie Barry, Jane Grimwood, Jeremy Schmutz, Jerry Jenkins, Alex Harkess, Bob VanBuren, Jim Leebens-Mack, Jill M. Farrant

Article acceptance date: 2 October 2025

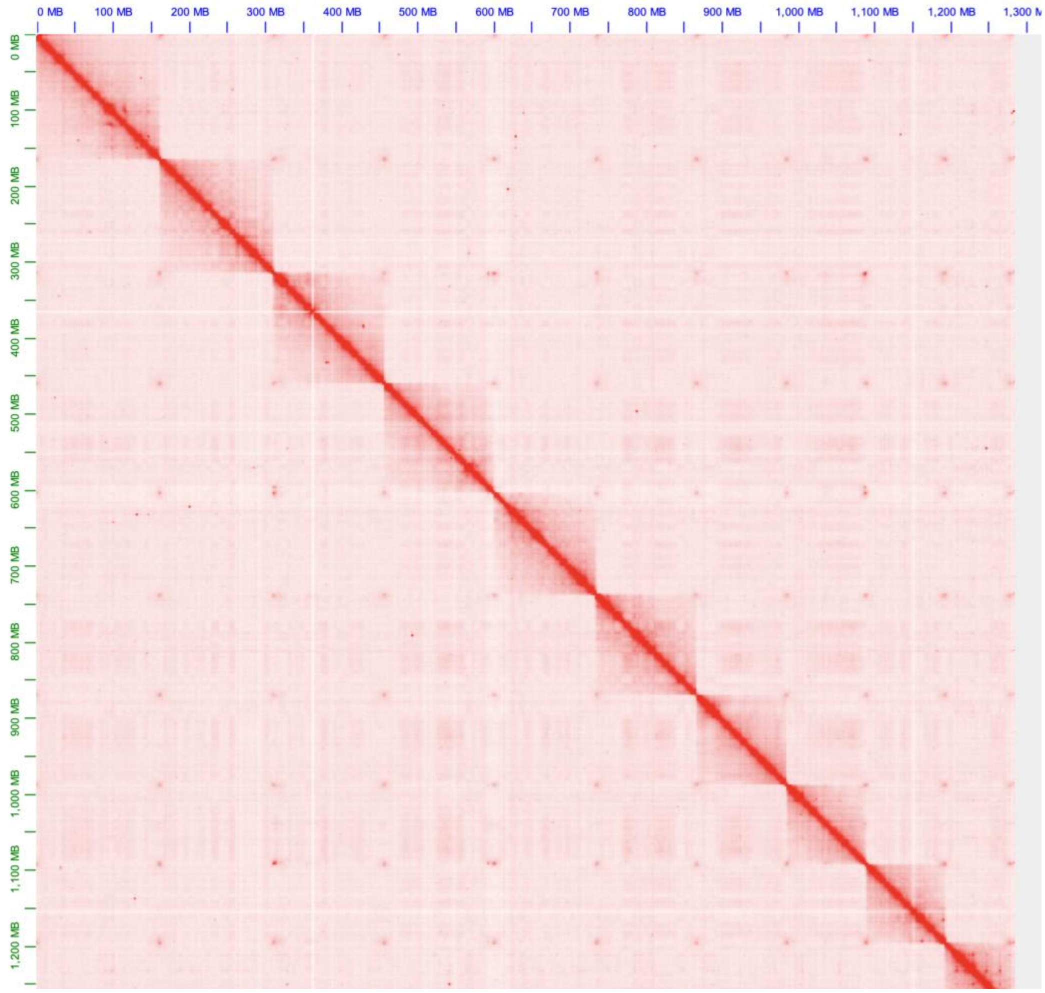

Supplementary Figure S1. Hi-C contact map showing the remarkably consistent 3D chromatin architecture visible in genome-wide Hi-C data.

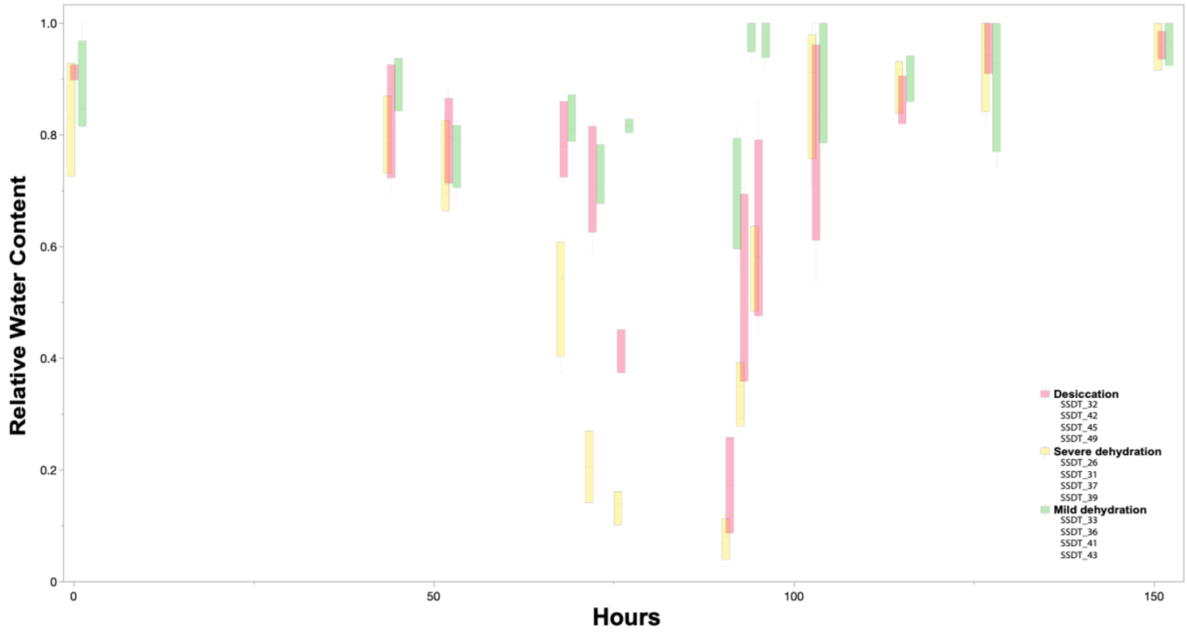

Supplementary Figure S2. Relative water content (RWC) of plants throughout the 6-day dehydration-rehydration time course measured in hours. Plant genotypes (e.g., SSDT_26) are listed and grouped / colored by their drying groups (“mild dehydration,” “severe dehydration,” or “desiccation”) based on minimum RWC.

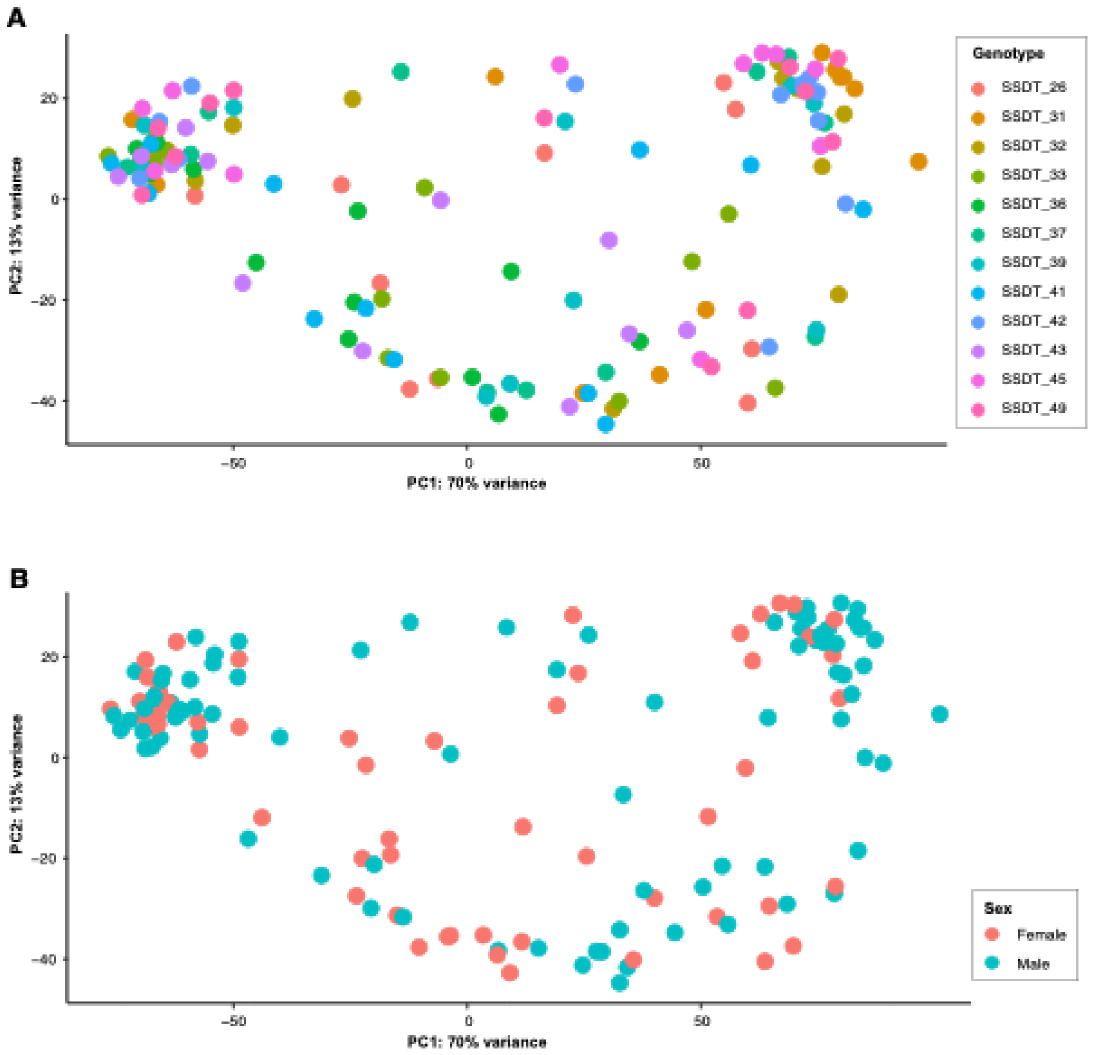

Supplementary Figure S3. Principal component analysis (PCA) of transcript abundance for all plants colored by genotype (A) and sex (B).

Construction of the scaffold assembly. A total of 6,672,299 PACBIO reads (42.99x per haplotype) were assembled using HiFiAsm+HIC assembler (Cheng et al., 2021), and formed the starting point of the version 1.0 release. The 1,592,906,904 Illumina fragment 2x150 reads (89.28x sequence coverage) was used for fixing homozygous snp/indel errors in the consensus. Chromosomes were scaffolded using the 1,047,438,958 2x150 (56.69x) HiC reads.

Table S1. Genomic libraries included in the *Myrothamnus flabellifolia (var. SSDT_37)* genome assembly and their respective assembled sequence coverage levels in the final release.
*Average read length of PACBIO reads.

**Library**	**Sequencing Platform**	**Average Read/Insert Size**	**Read Number**	**Assembled Sequence Coverage (x)**
JCHZ_1024	Illumina (2x150)	400	866,865,500	48.46
JCHZ_1027	Illumina (2x150)	400	726,041,404	40.82
KIQF	Illumina-HiC (2x150)	N/A	1,047,438,958	56.69
HSZWT	PACBIO	15,920*	6,672,299	42.99
**Total**		N/A	2,647,018,161	188.96

Table S2. PACBIO CCS library statistics for the libraries included in the *Myrothamnus flabellifolia (var. SSDT_37)* genome assembly and their respective assembled sequence coverage levels.

Cutoff	Number of Reads	Basepairs	Average Read Length	Coverage
0	6,672,299	110,486,722,577	15,920	42.99x
1,000	6,668,201	110,484,391,751	15,923	42.99x
2,000	6,662,596	110,475,853,380	15,927	42.99x
3,000	6,655,523	110,458,025,667	15,932	42.98x
4,000	6,645,888	110,424,127,530	15,939	42.97x
5,000	6,634,158	110,371,211,854	15,948	42.94x
6,000	6,620,629	110,296,607,955	15,958	42.92x
7,000	6,606,535	110,205,086,898	15,969	42.88x
8,000	6,592,903	110,102,827,301	15,979	42.84x
9,000	6,577,630	109,972,808,781	15,990	42.79x
10,000	6,555,731	109,763,856,821	16,006	42.71x
11,000	6,501,178	109,184,346,854	16,047	42.48x
12,000	6,179,853	105,452,613,236	16,290	41.03x
13,000	5,512,543	97,094,640,388	16,822	37.78x
14,000	4,747,524	86,764,891,354	17,486	33.76x
15,000	3,988,602	75,763,555,955	18,225	29.48x
16,000	3,282,195	64,819,629,728	19,011	25.22x
17,000	2,650,337	54,401,409,829	19,828	21.17x
18,000	2,104,845	44,862,857,820	20,660	17.46x
19,000	1,645,614	36,374,240,625	21,496	14.15x

Table S3. Summary statistics of the initial output of the HAP1 RACON polished HiFiAsm+HIC assembly. The table shows total contigs and total assembled basepairs for each set of scaffolds greater than the size listed in the left hand column.

Minimum Scaffold Length	Number of Scaffolds	Number of Contigs	Scaffold Size	Basepairs	% Non-gap Basepairs
5 Mb	84	84	1,097,947,157	1,097,947,157	100.00%
2.5 Mb	116	116	1,217,549,339	1,217,549,339	100.00%
1 Mb	144	144	1,265,106,648	1,265,106,648	100.00%
500 Kb	154	154	1,272,868,595	1,272,868,595	100.00%
250 Kb	160	160	1,275,059,470	1,275,059,470	100.00%
100 Kb	184	184	1,278,798,069	1,278,798,069	100.00%
50 Kb	370	370	1,290,360,047	1,290,360,047	100.00%
25 Kb	370	370	1,290,360,047	1,290,360,047	100.00%
10 Kb	370	370	1,290,360,047	1,290,360,047	100.00%
5 Kb	370	370	1,290,360,047	1,290,360,047	100.00%
2.5 Kb	370	370	1,290,360,047	1,290,360,047	100.00%
1 Kb	370	370	1,290,360,047	1,290,360,047	100.00%
0 bp	370	370	1,290,360,047	1,290,360,047	100.00%

Table S4. Summary statistics of the initial output of the HAP2 RACON polished HiFiAsm+HIC assembly. The table shows total contigs and total assembled basepairs for each set of scaffolds greater than the size listed in the left hand column.

**Minimum Scaffold Length**	**Number of Scaffolds**	**Number of Contigs**	**Scaffold Size**	**Basepairs**	**% Non-gap Basepairs**
5 Mb	91	91	1,067,419,368	1,067,419,368	100.00%
2.5 Mb	130	130	1,214,090,884	1,214,090,884	100.00%
1 Mb	163	163	1,269,161,900	1,269,161,900	100.00%
500 Kb	179	179	1,280,285,219	1,280,285,219	100.00%
250 Kb	191	191	1,284,416,048	1,284,416,048	100.00%
100 Kb	214	214	1,287,435,802	1,287,435,802	100.00%
50 Kb	333	333	1,295,198,434	1,295,198,434	100.00%
25 Kb	333	333	1,295,198,434	1,295,198,434	100.00%
10 Kb	333	333	1,295,198,434	1,295,198,434	100.00%
5 Kb	333	333	1,295,198,434	1,295,198,434	100.00%
2.5 Kb	333	333	1,295,198,434	1,295,198,434	100.00%
1 Kb	333	333	1,295,198,434	1,295,198,434	100.00%
0 bp	333	333	1,295,198,434	1,295,198,434	100.00%

Screening and Final Assembly Releases

Scaffolds that were not anchored in a chromosome were classified into bins depending on sequence content. Contamination was identified using blastn against the NCBI non-redundant nucleotide collection (NR/NT) and blastx using a set of known microbial proteins. Additional scaffolds were classified in the version 1.0 HAP1 release as redundant (unanchored scaffolds composed of >=95% 24mers >2x in all scaffolds) (138 scaffolds, 10.9 Mb), fungal (54 scaffolds, 3.2 Mb), repetitive (<=250 Kb scaffolds composed of >=95% 24mers >4x in >=5 Mb scaffolds) (21 scaffolds, 1.9 Mb), chloroplast (1 scaffolds, 158.8 Kb), and mitochondria (1 scaffolds, 21.3 Mb). Resulting final statistics for the HAP1 version 1.0 release are shown in Table S5.

Table S5. Final summary assembly statistics for the version 1.0 HAP1 chromosome scale assembly.

**Scaffold total**	12
**Contig total**	195
**Scaffold sequence total**	1,285.3 Mb
**Chromosome Sequence**	1,283.3 Mb
**Contig sequence total**	1,283.4 Mb (0.1% gap)
**Scaffold N/L50**	5 / 134.5 Mb
**Contig N/L50**	33 / 11.4 Mb

Additional scaffolds were classified in the version 1.0 HAP2 release as redundant (unanchored scaffolds composed of >=95% 24mers >2x in all scaffolds) (39 scaffolds, 4.3 Mb), fungal (28 scaffold, 2.1 Mb), repetitive (<=250 Kb scaffolds composed of >=95% 24mers >4x in >=5 Mb scaffolds) (16 scaffolds, 1.5 Mb), chloroplast (1 scaffolds, 158.8 Kb), and mitochondria (1 scaffolds, 21.3 Kb). Resulting final statistics for the HAP2 version 1.0 release are shown in Table S6.

Table S6. Final summary assembly statistics for the version 1.0 HAP2 chromosome scale assembly.

**Scaffold total**	10
**Contig total**	169
**Scaffold sequence total**	1,274.7 Mb
**Chromosome Sequence**	1,273.1 Mb
**Contig sequence total**	1,273.1 Mb (0.1% gap)
**Scaffold N/L50**	5 / 133.8 Mb
**Contig N/L50**	32 / 13.7 Mb
